# Dysregulated expression of *miR-140* and *miR-122* compromised microglial chemotaxis and led to reduced restriction of AD pathology

**DOI:** 10.1186/s12974-024-03162-z

**Published:** 2024-07-02

**Authors:** Chao Song, Shufang Li, Yingren Mai, Linpeng Li, Guoku Dai, Yuan Zhou, Xiaosheng Liang, Olivia Meilan Zou, Ya Wang, Libing Zhou, Jun Liu, Yi Zou

**Affiliations:** 1 School of Life Science and Technology, Jinan University, Guangzhou, Guangdong 510632 China; 2 Department of Neurology, Institute of Neuroscience, Key Laboratory of Neurogenetics and Channelopathies of Guangdong Province and the Ministry of Education of China, The Second Affiliated Hospital, Guangzhou Medical University, Guangzhou, Guangdong 510260 China; 3 Guangdong-Hongkong-Macau Institute of CNS Regeneration, Ministry of Education CNS Regeneration Collaborative Joint Laboratory, Jinan University, Guangzhou, Guangdong 510632 China

**Keywords:** Alzheimer’s disease, Microglial, Neuroinflammation

## Abstract

**Background:**

Deposition of amyloid β, which is produced by amyloidogenic cleavage of APP by β- and γ-secretase, is one of the primary hallmarks of AD pathology. APP can also be processed by α- and γ-secretase sequentially, to generate sAPPα, which has been shown to be neuroprotective by promoting neurite outgrowth and neuronal survival, etc.

**Methods:**

The global expression profiles of miRNA in blood plasma samples taken from 11 AD patients as well as from 14 age and sex matched cognitively normal volunteers were analyzed using miRNA-seq. Then, overexpressed *miR-140* and *miR-122* both in vivo and in vitro, and knock-down of the endogenous expression of *miR-140* and *miR-122* in vitro. Used a combination of techniques, including molecular biology, immunohistochemistry, to detect the impact of miRNAs on AD pathology.

**Results:**

In this study, we identified that two miRNAs, *miR-140-3p* and *miR-122-5p*, both targeting ADAM10, the main α-secretase in CNS, were upregulated in the blood plasma of AD patients. Overexpression of these two miRNAs in mouse brains induced cognitive decline in wild type C57BL/6J mice as well as exacerbated dyscognition in APP/PS1 mice. Although significant changes in APP and total Aβ were not detected, significantly downregulated ADAM10 and its non-amyloidogenic product, sAPPα, were observed in the mouse brains overexpressing *miR-140*/*miR-122*. Immunohistology analysis revealed increased neurite dystrophy that correlated with the reduced microglial chemotaxis in the hippocampi of these mice, independent of the other two ADAM10 substrates (neuronal CX3CL1 and microglial TREM2) that were involved in regulating the microglial immunoactivity. Further in vitro analysis demonstrated that both the reduced neuritic outgrowth of mouse embryonic neuronal cells overexpressing *miR-140*/*miR-122* and the reduced Aβ phagocytosis in microglia cells co-cultured with HT22 cells overexpressing *miR-140*/*miR-122* could be rescued by overexpressing the specific inhibitory sequence of *miR-140*/*miR-122* TuD as well as by addition of sAPPα, rendering these miRNAs as potential therapeutic targets.

**Conclusions:**

Our results suggested that neuroprotective sAPPα was a key player in the neuropathological progression induced by dysregulated expression of *miR-140* and *miR-122*. Targeting these miRNAs might serve as a promising therapeutic strategy in AD treatment.

**Supplementary Information:**

The online version contains supplementary material available at 10.1186/s12974-024-03162-z.

## Background

Alzheimer’s disease (AD) is a progressive neurodegenerative disorder that causes the most common form of dementia in the elderly [1]. The underlining pathological events in brains are characterized by the senile plaque and neurofibrillary tangles made by accumulated beta-amyloid (Aβ) and hyperphosphorylated tau protein, respectively [2]. Aβ proteins were produced by amyloidogenic proteolysis of amyloid precursor proteins (APP), which are transmembrane proteins displaying G protein-dependent receptor activity [3]. APP can be cleaved by α- or β-secretase to produce soluble APPα (sAPPα) or APPβ, the C-terminal fragment of the latter further cleaved by γ-secretase to release small Aβ peptides ranging from 38 to 43 amino acids [4]. The fragment Aβ42 tend to form self-aggregates, which are believed to be neurotoxic and are the predominant forms found in AD amyloid plaques [5]. It is currently proposed that soluble Aβ oligomers, rather than monomers or fibrils, are the cytotoxic species to induce neuroinflammation, oxidative stress, synaptic dysfunction, and eventually neuronal loss [6, 7]. The majority of brain-derived Aβ, up to 60%, is cleared in the periphery via receptor-mediated transport across BBB as well as via interstitial pathway into CSF [8]. The Aβ peptides can also be up taken by phagocytosis of microglia and astrocytes, which also degrade Aβ by secreting a number of proteases [9]. However, Aβ peptide also displayed neuroprotective function at low concentrations [10, 11]. Therefore, the life-long proper Aβ production is dependent on the precise adjustment of Aβ generation and clearance, both of which are fundamental therapeutic strategies regarding AD treatment [12–14].

Unlike the amyloidogenic peptides, sAPPα appeared to be neuroprotective, although the detailed downstream pathway remained unclear. It was shown to promote neurite outgrowth, enhance synaptogenesis, and displayed neurotrophic effect to prolong neuronal survival in deleterious environment [15, 16]. ADAM10, a member of ADAM (a disintegrin and metalloprotease) family, is the main α-secretase that cleaves APP in brain [17, 18]. The expression of ADAM10 is intricately regulated and among them, the transcriptional regulation by *all-trans* retinoic acid (*at*RA) has been well studied [19]. RA-induced ADAM10 expression has been shown to be beneficial in AD models both in vitro and in vivo [20, 21]. Diminishing ADAM10 activities, via mutations in the functional domain of ADAM10 in AD mice, lead to increased amyloidogenic processing of APP and significantly reduces neurogenesis [13].

Here we reported that two miRNAs, *miR-140-3p* and *miR-122-5p* (referred to as *miR-140* and *miR-122* in this work), both targeting ADAM10, were upregulated in the blood plasma of AD patients as well as in the brains of APP/PS1 mice. Overexpression of *miR-140* and *miR-122* in mouse brains significantly downregulated ADAM10 and induced cognitive impairment in wild type mice as well as exacerbated cognitive dysfunction in APP/PS1 mice. Treatment of *at*RA significantly improved cognition in mice overexpressing *miR-140* and *miR-122* via upregulating the expression of ADAM10. Reduced microgliosis and microglia activation were observed in the hippocampi of APP/PS1 mice overexpressing *miR-140*/*miR-122*. These inert microglia cells displayed reduced chemotaxis and phagocytosis and thus exacerbated neuritic dystrophy in AD mice. The neuritic dystrophy in cultured embryonic neuronal cells overexpressing *miR-140*/*miR-122* and the inert microglia co-cultured with neuronal cells overexpressing *miR-140*/*miR-122* could be rescued by *at*RA treatment as well as by addition of sAPPα but not by blocking with anti-Aβ. In addition, overexpression of the specific inhibitory *miR-140*/*miR-122* TuD significantly enhanced neurite outgrowth and microglial phagocytosis in vitro. Taken together of the results in this study, we proposed that reduced neuroprotective sAPPα played a key role in regulating the neuropathological progression associated with dysregulated *miR-140* and *miR-122* expression in AD mice. Targeting these miRNAs might serve as a potential therapeutic strategy in AD treatment.

## Materials and methods

### Reagents

DMEM, fetal bovine serum (FBS), and Lipofectamine™ 2000 transfection reagents were purchased from Invitrogen. 1,4-dithiothreitol (DTT), dimethyl sulfoxide (DMSO), Triton X-100, 4% paraformaldehyde, bovine serum albumin, ethanol, Hoechst 33,342, RIPA buffer, phosphatase inhibitor cocktail, *all-trans* retinoic acid, corn oil, thioflavine S, penicillin, and streptomycin were from Sigma. Blue RangeTM prestained protein molecular marker and BCA Protein Assay Kit were from Thermo Fisher Scientific. Recombinant Human Amyloid Precursor Protein alpha was purchased from Antibodies.com (Cambridge, UK). Anti-ADAM10 antibody (Cat. No. ab124695, 1:5000) and anti-CX3CL1 antibody (Cat. No. ab25088, 1:1000) were purchased from Abcam (Cambridge, UK). Anti-APP antibody (Cat.No.2045, 1:2000) and was purchased from Cell Signaling Technology (Beverly, MA, USA). Anti-6E10 antibody (Cat. No. 803014, 1:5000) was purchased from Biolegend (San Diego, CA, USA). Anti-beta Amyloid antibody (MOAB-2, Cat. No. 13075, 1:500) was purchased from Novus Biologicals (Littleton, CO, USA). Anti-Iba1 antibody (Cat. No. 019-19741, 1:1000) was purchased from FUJIFILM Wako Pure Chemical Corporation (Osaka, Japan). Anti-TREM2 antibody (Cat.No.27599-1-AP, 1:1000) was purchased from Proteintech (Chicago, IL). Anti-CX3CR1 antibody (Cat.No.38,481, 1:1000) was purchased from Signalway Antibody (SAB, College Park, Maryland, USA). HRP-conjugated Affinipure Goat Anti-Mouse IgG (H + L) (Cat. No. SA00001-1, 1:3000), HRP-conjugated Affinipure Goat Anti-Rabbit IgG (H + L) (Cat. No. SA00001-2; 1:3000) were purchased from Proteintech (Chicago, IL). Alexa Fluor 647-conjugated Affinipure Goat Anti-Mouse IgG (H + L) (Cat. No. 115-605-003, 1:1000), Cy3-conjugated AffiniPure F(ab’)2 Fragment Goat Anti-Rabbit IgG (H + L) (Cat. No. 111-166-144, 1:1000) was purchased from Jackson ImmunoResearch Laboratories (West Grove, PA, USA). *MiR-140-3p* and *miR-122-5p* mimics/ scramble RNA were synthesized by GenePharma (Shanghai, China). The *miR-140-3p* mimics and *miR-122-5p* mimics were double-stranded (5ʹ-UACCACAGGGUAGAACCACGG-3ʹ and 5ʹ-UGGAGUGUGACAAUGGUGUUUG-3ʹ, respectively). The scramble RNAs was double-stranded RNA (5ʹ- UUCUCCGAACGUGUCACGUTT-3ʹ).

### Participants and sample collection

The participants, aged between 60-90-year-old, were recruited from the Department of Neurology, the Second Affiliated Hospital of Guangzhou Medical University and the study were approved by the ClinicalTrials.gov (ID: NCT03653156). All patients gave written informed consent. All participants underwent the Mini-Mental State Examination (MMSE) and those scored > = 27 was considered as cognitive normal controls. Participants with a MMSE score < = 26 went through additional tests of cerebrospinal fluid Aβ and p-Tau. Diagnosis of AD was made in accordance with the 2018 NIA-AA Pathological Diagnostic Guidelines. Aβ42 < 550 pg/mL or Aβ42/Aβ40 ≤ 0.05 (Amyloid-beta (1–40) High Sensitive ELISA and Amyloid-beta (1–42) High Sensitive ELISA, Cat. No. RE59781, Cat. No. RE59791, IBL International GmbH., Hamburg, Germany), p-Tau > 50 pg/mL (phosphoTAU ELISA, Cat. No. 30,121,609), and t-Tau > 399 pg/mL (hTau total ELISA, Cat. No. RE59631) was considered ATN positive, and these participants were included in the study providing no presence of other brain diseases. The final cohort consisted of 11 AD and 14 cognitive normal subjects. 2 mL peripheral blood from the cubital vein were collected using EDTA anticoagulation tubes (Cat. No.367863, Becton, Dickinson and Company, Franklin Lakes, NJ, USA) in the early morning after refraining from eating and drinking for eight hours and were allowed to sit for 30 min at room temperature. Plasma samples were collected from the supernatant after centrifugation at 3,000 rpm for 10 min and stored in 100 µL aliquots at -80℃ before use.

### Animals

4-month-old male APP/PS1 mice and control C57BL/6 mice were purchased from The Experimental Animal Center of Guangdong (Guangzhou, China) and bred in the Laboratory Animal Management Center of the Jinan University in a pathogen free facility. All mice were housed at temperature (21–25℃) under 12 h- light/dark cycle with free access to water and food. All animal experiments were approved by the Institutional Animal Care and Use Committee of Jinan University (IACUC-20230625-02). For *at*RA treatment, *at*RA was dissolved in DMSO and diluted in corn oil to make the final concentration is 5% DMSO (v/v). *at*RA (20 mg/kg) was applied by intraperitoneal injection three times per week. The control mice were mock treated with vehicles. Administration of *at*RA was initiated 24 h after the stereotactic injection and continued for 8 weeks. All animals were sacrificed using CO_2_ asphyxiation before the tissue samples were collected.

### Stereotactic injection

Mice were anesthetized with 2% pentobarbital sodium (50 mg/kg) and placed in the stereotactic platform (Harvard Apparatus, Holliston, MA). AAV9-*miR140*-*miR122*-eGFP or AAV9-Scr-eGFP (4 × 10^10^ genome copies × 2) were injected into both sides of the lateral ventricles (posterior: +0.3 mm; mediolateral: ± 1.0 mm, dorsal: -2.0 mm) at a rate of 0.4 µL/min using a micro-drive arm of the micropump (KDS Legato™ 130 micro-pump, KD Scientific Inc., Holliston, MA) attached to a Hamilton 5 µL syringe (87930 Hamilton, Reno, NV). The needle was held in place for 5 min to prevent leakage before retraction after each injection. Mice were recovered in a warming cabinet before returning to their home cage.

### Behavioral assessment

Mice were brought to the testing room and allowed to acclimate for 1 h before assessment. A Y-maze apparatus composed of three enclosed arms (35 cm long × 15 cm high × 5 cm wide) oriented at 120° angles from each other was used for the Y maze test to assess short-term spatial working memory. Mice were introduced at the end of one of the arms and allowed for 8-min free exploration. A spontaneous alternation was defined as consecutive entries into three arms. Morris water maze (MWM) test was performed using a circle pool with a diameter of 120 cm diameter and a depth of 60 cm filled with water at room temperature (24℃). The pool was virtually divided into four quadrants and the escape platform (10 cm diameter) was placed in one of the quadrants submerged 1 cm under water level. The test started by placing the mouse in the pool at different quadrants in each training and the mouse were allowed to find the platform within a maximum trial time of 90 s. The trial was completed if the mice reached the platform within that time and was allowed to remain on the platform for 10 s before returning. If the mouse was unable to find the platform within 90 s, it was guided to the platform and allowed to stay for 10 s. The training was performed on 4 successive days with two trials per day, with one hour gap between. The probe trial was performed on the fifth day with the platform removed from the pool. Initiation started from the farthest direction from the position of platform used during training. The time spent in the platform quadrant and the number of crossing the platform location were calculated. The activities of the mice in all behavioral tests were recorded using a video tracking camera and analyzed using ANY-maze software (Stoelting Co. USA).

### miRNA sequencing data processing

Total RNAs from plasma samples were extracted using mirVana miRNA Isolation Kit (Cat.No.AM1561, Ambion, Austin, TX, US) following the manufacturer’s instructions. The RNA concentrations were determined by the absorbances at 260 nm using NanoDrop ND-2000 spectrophotometer (Thermo Fisher Scientific, Waltham, MA, USA). Libraries for sequencing were prepared using 10 ng of total RNA using QIAseq miRNA Library Kit (Cat.No. 331502, Qiagen, Germantown, MD, USA). In brief, adapters are ligated sequentially to the 3’ and 5’ ends of miRNAs, followed by universal cDNA synthesis, cDNA cleanup, library amplification and library cleanup. Constructed libraries were quantified and validated using Qubit 2.0 Fluorometer (Life Technologies, Carlsbad, CA, USA) and 2400 bioanalyzer (Agilent Technologies, USA) system, and were then sequenced on an Illumina NovaSeq 6000 system using PE150 model (Shanghai Biotechnology Corporation) at an average read depth of 20–35 million reads per sample.

Raw reads were processed with fastx (version 0.0.13, http://hannonlab.cshl.edu/fastx_toolkit/) to remove adaptors, low-quality reads, and reads shorter than 10 nt to obtain clean reads. The clean reads (18–40 nts) were aligned to miRNAs in miRbase20.0 (http://www.mirbase.org/) using Bowtie2 software (version 2.4.4) [22, 23]. Read counts were normalized using the trimmed mean of M-values (TMM) method, and then referenced the sequencing depth to get values of transcripts per million (TPM) [24, 25]. In addition, for unannotated miRNAs, we used miRCat pipeline in the UEA sRNA workbench (version 4.4) to evaluate potential novel miRNA candidates according to miRNA precursor hairpins [26].

### Bioinformatic analysis

miRNAs were further filtered by expression level to exclude low expression genes using the ‘filterByExpr’ function and a count value > = 10 in over 50% of the samples in each group (*n* > 7 in AD group and *n* > 6 in control group) was used as cut-off. The gene counts were normalized by the method of trimmed mean of M-values using the ‘calcNormFactors’ function of edgeR. Principle component analysis of the normalized miRNA expression data after filtering were performed using the ‘prcomp’ function and the differentially expressed miRNAs (DEMs) between the controls and AD patients was analyzed using the ‘glmQLFit’ function of edgeR [27, 28]. The fold-change of at least 2 with adjusted p-value (FDR) < 0.05 was used as the cutoffs. The heatmap of DEMs was generated using ‘pheatmap’ package and the relation between groups was depicted by hierarchically clustered according to *Pearson* correlation and average linkage. The volcano plot, which the x-axis represents the log2-transformed fold change and the y-axis the log10-transformed p-value was constructed by using ‘ggplot2’ package The above process is all implemented in R (version 4.2.1). Comprehensive Automatically Mined Database of Human Diseases (Malacards) was used to search cognitive dysfunction related genes for a gene list. Online analysis tools of TargetScan (version 8.0) and microT-CDS (version 5.0) were used for miRNA target gene prediction. ‘VennDiagram’ package of R software (version 4.2.1) was employed to depict the intersections of target genes of DEMs and cognitive dysfunction related genes. The miRNA-mRNA regulatory network was visualized by using Cytoscape (version3.9.1). Functional annotation of Gene Ontology (GO, Biological Process, Cellular Component, Molecular Function) and Kyoto Encyclopedia of Genes and Genomes (KEGG) pathway enrichment analysis of cognitive dysfunction related miRNA targets were performed using DAVID (v2022q2, https://david.ncifcrf.gov/) based on total miRNA targets as a population background.

### miRNA mimics, recombinant AAV vectors and *miR-140-miR-122* TuD expression vectors

Mimics of *miR-140* and *miR-122*, and scramble miRNA were purchased from GenePharma (Shanghai, China). The sequences of *miR-140-3p* and *miR-122-5p* were obtained from miRBase database (www.mirbase.org). The sequence of the stem loop structure of these two miRNAs (Table S4) were cloned into the expression plasmids GV412 (Genechem, Shanghai, China). The AAV vector GV412 contains CMV promoter and encoding eGFP. The 293T cells were transfected with the recombinant AAV vectors, pHelper and pAAV9 rep-cap using Lipofectamine 2000. After transfection for 48 h, cells were harvested and lysed by four freeze-thaw cycles in dry ice and 37℃ water baths for collect the virus. The lysed incubation with benzonase (Sigma Aldrich) at 37℃ for 30 min to concentrate virus. Then, the virus was purified by the CsCl gradient ultracentrifugation and were further concentrated on Amicon-15 Centrifugal Filters (Merck Millipore, Burlington, MA, USA). AAV genome copies were titrated using quantitative PCR (qPCR).

DNA sequences encoding tough decoy RNAs targeting both *miR-140-3p* and *miR-122-5p*, designed according to Haraguchi *et al*., were synthesized by Sangon Biotech (Shanghai, China) and were cloned into the *NheI*/*HindIII* sites of pcDNA3.1 [29]. The *miR-140*-*miR-122*-TuD contains two tandem stem-loop structures, each of which contains either the *mmu-miR-140-3p* or *mmu-miR-122-5p* binding sites flanked by an 18-bp long stem and a 26-bp stem-loop, respectively. A 4-nt long mismatch was inserted into each of the miRNA-binding sites between 10 and 11 from the 3’-end to avoid complete paring. The schematic representation and sequence of the *miR-140*-*miR-122*-TuD is shown in Fig. 2. The sequences of all constructs were verified by sequencing (Sangon Biotech, Shanghai).

**Fig. 1:**
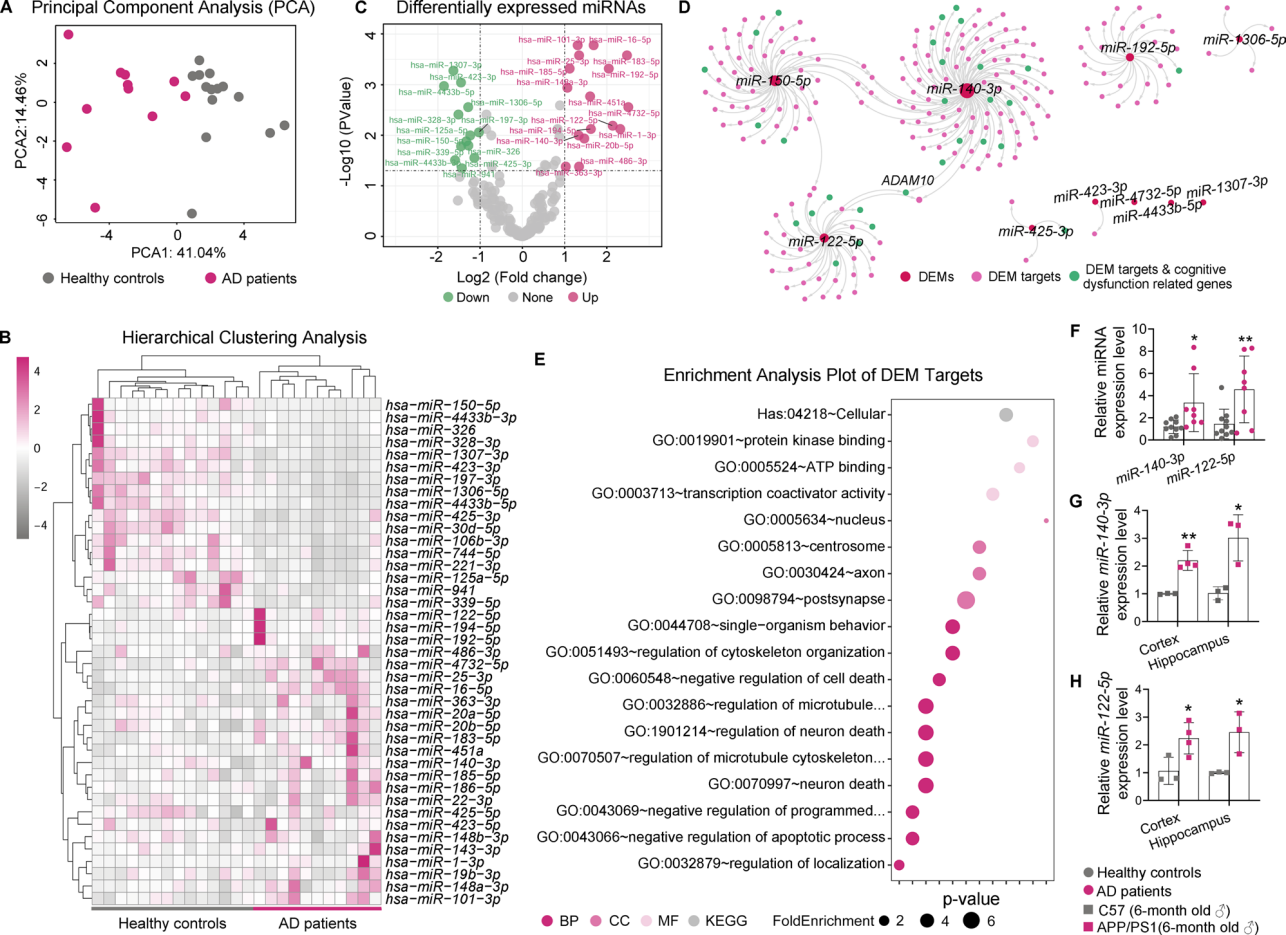
miRNA profiling of the blood plasma of AD patients and cognitively normal controls. (**A**). PCA analysis of the normalized expression values of plasma miRNA, showing the separation between AD patients and controls on the first principal component. (**B**). Heat map showing the clustering of differentially expressed miRNAs. (**C**). Volcano plot displaying the fold change and p value of DEMs, with the labeling of all DEMs with |log2-fold change| >1 and FDR adjusted *p* < 0.05. (**D**). miRNA–mRNA regulatory network visualization for the enrichment analysis of DEMs. The size of miRNA nodes indicated the number of their target genes. Red circles represent DEMs and pink/green circles are their target genes, which green circles also are cognitive dysfunction related genes. (**E**) Gene Ontology biological process terms and Kyoto Encyclopedia of Genes and Genomes pathways in enrichment analysis of DEMs targets. Dot size represents the fold enrichment. The color of each dot represents the category of ontology, and the X-axis represents the p-value. (**F**). Quantitative RT-PCR validation of the expression of *miR-140* and *miR-122* in the plasma of AD patients and controls. The results were presented as means ± SD (*n* ≥ 9). (**G**-**H**). Quantitative RT-PCR data showing the expression of *miR-140* (**G**) and *miR-122* (**H**) in the cortex and the hippocampi of C57BL/6J mice and the APP/PS1 mice, respectively. The results were presented as means ± SD (*n* ≥ 3). *p < 0.05, ** *p* < 0. 01. Statistical analyses were performed using a two-tailed unpaired *Student’s t*-test

**Fig. 2:**
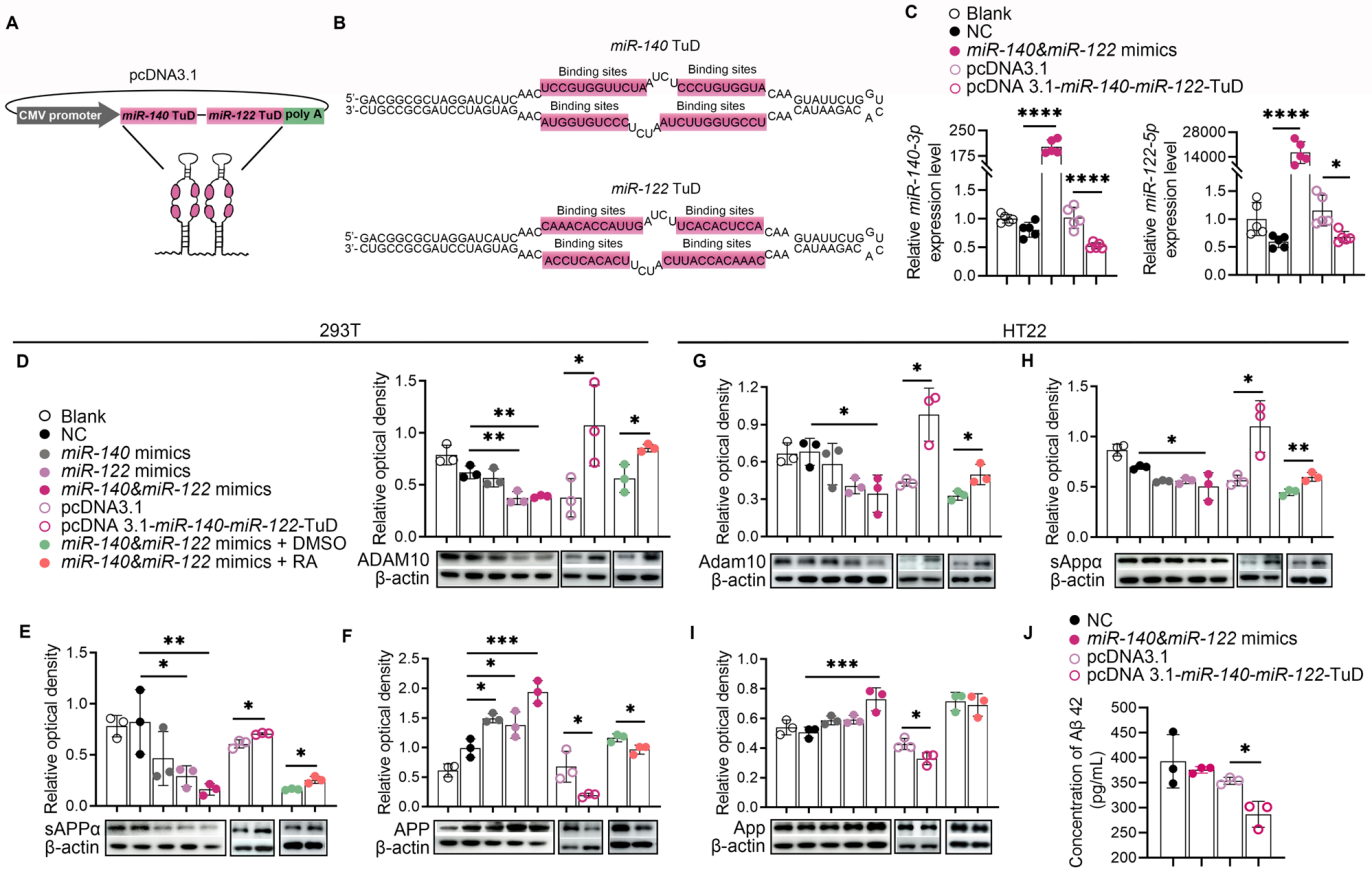
Dysregulated expression of *miR-140* and *miR-122* affected sAPPα production via targeting ADAM10. (**A**). The specific inhibitory sequence of *miR-140*-*miR-122*-TuD containing two tandem stem-loop structures was cloned into the *NheI*/*HindIII* sites of pcDNA3.1. (**B**). The sequences of *miR-140*-*miR-122*-TuD, with each of the stem -loop contains two miRNA-binding sites flanked by an 18-bp long stem and a 26-bp stem-loop, respectively. A 4-nt long mismatch was inserted into each of the miRNA-binding sites between 10 and 11 from the 3’-end to avoid complete paring. (**C**). Quantitative real-time PCR showing significantly upregulated or downregulated *miR-140* and *miR-122* expression levels in 293T cells transfected with mimics of *miR-140* and *miR-122* or pcDNA3.1-*miR-140*-*miR-122*-TuD. Untransfected cells and cells transfected with scramble miRNA and pcDNA3.1 were used as controls. The results were presented as means ± SD (*n* = 5). *p < 0.05, ****p < 0.0001. Statistical analyses were performed using One-way ANOVA. (**D**-**I**). Cultured 293T cells (**D**-**F**) and HT22 cells (**G**-**I**) were transfected with: (1) vehicle only; (2) scramble miRNA; (3) mimics of *miR-140*; (4) mimics of *miR-122*; (5) mimics of *miR-140* and *miR-122*; (6) pcDNA3.1; (7) pcDNA3.1-*miR-140*-*miR-122*-TuD; (8) mimics of *miR-140* and *miR-122* + mock treatment; (9) mimics of *miR-140* and *miR-122* + 2 µM *at*RA. The control cells were treated with vehicles. (**D**-**F**). Western blot analysis and densitometric quantification of the relative expression of ADAM10 (**D**), sAPPα (**E**) and APP (**F**) in the transfected 293T cells as indicated. The results were presented as means ± SD (*n* = 3). *p < 0.05, **p < 0.01, ***p < 0.001. Statistical analyses were performed using One-way ANOVA or two-tailed unpaired *Student’s t*-test. (**G**-**I**). Western blot analysis and densitometric quantification of the relative expression of ADAM10 (**G**), sAPPα (**H**) and APP (**I**) in the transfected HT22 cells as indicated. The results were presented as means ± SD (*n* = 3). *p < 0.05, **p < 0.01, ***p < 0.001. Statistical analyses were performed using One-way ANOVA or two-tailed unpaired *Student’s t*-test. (**J**). ELISA analysis of the soluble Aβ42 monomer in the cultured HT22 cells transfected with scramble miRNA, mimics of *miR-140* and *miR-122*, pcDNA3.1 and pcDNA3.1-*miR-140*-*miR-122*-TuD. The results were presented as means ± SD (*n* = 3). *p < 0.05, statistical analyses were performed using One-way ANOVA

### RNA extraction and quantitative real time-PCR (qRT-PCR)

For isolation of miRNAs from blood plasma, the miRNAs were isolated from 100 µL of blood plasma using the miRNeasy Serum/Plasma Kit (Cat.No. 217184, Qiagen, Germantown, MD, USA) according to the manufacturer’s protocols. 1 pmol synthetic *Caenorhabditis elegans* miRNA *cel-miR-39* was used as an internal standard and added to each sample. The final RNA was eluted with 10 µL of nuclease-free water and 3.75 µL RNA of each sample was reverse transcribe using the Mir-X miRNA First-Strand Synthesis Kit (Cat.No. 638313, Takara, Ohtsu, Japan). The primer pairs consist of a common mRQ 3’ primer and one of the specific 5’ primers (*hsa-miR-140-3p*: 5’-AAGTTGCATACCACAGGGTAGA-3’, *hsa-miR-122-5p*: 5’-AACACGCTGGAGTGTGACAA-3’, *cel-miR-39*: 5’-ACCGAGGTTCACCGGGTGTAA-3’) were used to amplify *hsa-miR-140-3p*, *hsa-miR-122-5p* and *cel-miR-39*, respectively.

For RNA isolation from mouse tissues, the mouse hippocampal and cortical tissues were first homogenized and the total RNA was extracted using Trizol-chloroform and isopropanol. 1 µg of total RNA was reverse transcribe using specific stem-loop primer (*miR-140-3p*: 5’-GTCGTATCCAGTGCAGGGTCCGAGGTATTCGCACTGGATACGACCCGTGG-3’, *miR-122-5p*: 5’-GTCGTATCCAGTGCAGGGTCCGAGGTATTCGCACTGGATACGACCAAACA-3’, *snRNA-U6*: 5’-CGCTTCACGAATTTGCGTGTCAT-3’) to synthesize cDNAs using the PrimeScript RT reagent Kit with gDNA Eraser (Cat. No. RR047A, Takara, Kusatsu, Japan). The primer pairs (5’-AAGTTGCATACCACAGGGTAGA-3’ and 5’-CAGTGCAGGGTCCGAGGT-3’, 5’-AACACGCTGGAGTGTGACAA-3’ and 5’-CAGTGCAGGGTCCGAGGT-3’) were used to amplify *mmu-miR-140-3p* and *mmu-miR-122-5p*, respectively. The primer pairs (5’-CCGTTCCTGCGTTCTTAT-3’ and 5’- ACTGGTTGCCCTTGGTAG-3’, 5’-ATCTGACCTTCCTGCCCTCCAC-3’ and 5’-ACCCAAGAGCACACACGACATTC-3’) were used to amplify *Chi3l1* and *H2-Ea* cDNA. cDNA of *Gapdh* was amplified using the primer pairs (5’-GGCAAATTCAACGGCACAGTCAAG-3’ and 5’-TCGCTCCTGGAAGATGGTGATGG-3’) and was used as the internal reference of gene expression. Quantitative real-time PCR was performed using TB Green Premix Ex TaqII (Tli RNaseH Plus) (Cat. No. RR820A, Takara, Kusatsu, Japan) on an Applied Biosystems Quanstudio3 platform (Applied Biosystems, Waltham, MA). The relative expression of genes was calculated using the 2^−ΔΔCt^ formula and were normalized using internal controls of *cel-miR-39*, *U6* and *Gapdh*, respectively.

### Western blot

The cells and mouse brain tissues were lysed in RIPA buffer (Sigma) containing PMSF and phosphatase inhibitor cocktail. Protein concentration was determined using BCA Protein Assay Kit (Thermo Fisher Scientific). 20 µg proteins of each sample were separated on 10% SDS-PAGE gel and were then transferred onto PVDF membranes (BioRad). The membranes were blocked with 5% skim milk in 0.1% TBS-Tween 20 for 1.5 h under room temperature and probed with primary antibodies overnight at 4℃. The membranes were washed three times with 0.1% TBS-Tween 20 and probed with HRP labeled secondary antibodies for 2 h at RT. The immunoblots were visualized using Amersham Imager680 (GE Healthcare Bio-Sciences Corp, Piscataway, NJ) and the bands intensities were quantified by densitometric analysis with ImageJ software (ImageJ 1.8.0).

### Quantification of Aβ plaques, number of microglia cells, Iba1-positive areas, microglia coverage and dystrophic neurites using immunohistochemistry

Mice were anesthetized with 2% pentobarbital sodium (50 mg/kg) and perfused with 0.9% saline. The mouse brain was removed in ice and fixed in 4% paraformaldehyde (PFA) overnight at 4℃, followed by graded sucrose solutions (20%, 30% in PBS) at 4℃. Hemisphere brains were embedded in O.C.T. compound and store at -80℃. 15-µm and 30-µm-thick serial coronal sections of hippocampus region were prepared with a cryostat microtome (CM1950, Leica). For immunolabeling of Aβ deposits, 15-µm-thick sections were washed with PBS and permeabilized with 0.1% Triton X-100 in PBS for 15 min at room temperature (RT). After blocking with 10% goat serum in PBS at RT for 1 h, the tissue sections were subjected to overnight incubation at 4℃ with primary antibodies (MOAB-2, 1:1000, Cat. No. 13075, Novus Biologicals, Littleton, CO, USA and anti-Iba1, 1:1000, Cat. No. 016-20001, Wako Chemical, diluted in PBS + 1.5% goat serum), respectively. The sections were then washed with 0.1% Triton X-100 in PBS 5 min for 3 times, followed by incubation with Alexa Fluor 647-conjugated Affinipure F(ab′ )₂ Goat Anti-Mouse IgG (1:1000, Cat. No. 115-605-003, Jackson ImmunoResearch Laboratorie) and Cy3-AffiniPure F(ab′ )₂ fragment Goat anti-Rabbit IgG (1:1000, Cat. No. 111-166-144, Jackson ImmunoResearch Laboratorie) for 1 h at room temperature, respectively. The nuclei were counterstained using Hoechst 33342 (1:10000, Cat. No. 14533, Sigma-Aldrich) in Fluoromunt™ Aqueous Mounting Medium (Cat. No. F4680; Sigma-Aldrich) and coverslipped before imaging. The unilateral hippocampal regions of three consecutive coronal sections visualized using Olympus FV3000 confocal microscope and the images were captured at 10× magnification. The number and size of MOAB-2 positive Aβ plaques as well as Iba1-positive areas were quantitated using ImageJ.

30-µm-thick tissue sections were used for microglial enveloping analysis. The tissue sections were sequentially permeabilized with 0.5% Triton X-100 in PBS for 1 h at RT, blocked in 10% goat serum + 0.5% Triton X-100 in PBS for 1 h at RT, and probed with indicated primary antibodies (anti-Iba1, 1:100, Cat. No. sc-32,725, Santa cruz biotechnology, anti-lamp1, 1:800, Cat. No. ab24170, Abcam, diluted in 0.5% Triton X-100 containing 1.5% goat serum) overnight at 4℃. The sections were then incubated with Goat Anti-Mouse IgG (1:1000, Cat. No. 115-605-003, Jackson ImmunoResearch Laboratorie), and Cy3-AffiniPure F (ab′) ₂ fragment Goat anti-Rabbit IgG (1:1000, Cat. No. 111-166-144, Jackson ImmunoResearch Laboratorie) for 2 h at RT, respectively. Briefly washed 3X in PBS, the sections were stained with 1% Thioflavine S for 8 min at RT, followed by washing in 70% ethanol for 4 min and rinsing with water. The slides were mounted in Fluoromunt™ Aqueous Mounting Medium (Cat. No. F4680; Sigma-Aldrich) and coverslipped before imaging. The unilateral hippocampal fields of three consecutive coronal sections were visualized using Olympus FV3000 confocal microscope and the z-stacked images were captured using a 40 × objective at 1024 × 1024-pixel resolution at 3 μm z-step. The number and size of ThioS-positive Aβ plaques as well as the number of microglial cells within 25 μm radius from the center points of the Aβ plaques were quantified using ImageJ. The neuritic dystrophy associated with compact amyloid plaques was measured using ImageJ according to the method described by Condello et al., 2015 [30]. A Z-projection of three optical slices through the center of the plaque was generated for each of the plaques and was used to calculate microglia coverage as well as the dystrophic neuritic area. The colocalization of the plaques and Iba1-positive areas was recognized using ImageJ. The intersections between the colocalized areas and the plaque perimeter, which was defined using ImageJ, were identified manually for each of the plaque. The proportion of the summed arcs of the perimeter with Iba1 colocalization was defined as microglia coverage. To compare the dystrophic neurite formation in areas with or without microglia coverage, the angles used to define microglia coverage were further extended to the limit of Lamp1-positive areas (plaque area excluded), which were recognized using ImageJ. The Lamp1-positive areas within this extended selection as well as the Lamp1-positive areas outside the selection were calculated, respectively and were normalized to their respective angular degrees. The significance of correlation between microglia coverage and Lamp1-positive areas in different size of Aβ plaque was tested.

### Mouse embryonic primary cortical cell culture

Primary cortical tissues were isolated from E15.5 C57BL/6J embryo [31, 32]. The pregnant female mice (E15.5) were euthanized with CO_2_ and the embryos were collected in ice-cold Hank’s balanced salt solution (HBSS, Invitrogen). The cortical tissues of the embryos were dissected in fresh pre-chilled HBSS under a stereomicroscope, followed by digestion in 0.25% trypsin at 37 °C for 15 min. The cortical tissues were then resuspended in plating medium (BME medium + 10% FBS + 0.45% glucose (20%) + 1% sodium pyruvate + 1% glutamine + 1% Penicillin/streptomycin) and triturated with care using a pasteur pipettes until a single-cell suspension was obtained. Dissociated cells were inoculated in six-well plates that were coated with poly-L-lysine (1 mg/mL) and were allowed for settle in an incubator filled with 5% CO_2_ at 37 °C for 4 h. The medium was then replaced with neuron medium (Neurobasal medium + 2% B27 + 1% glutamine + 0.5% penicillin/streptomycin). The cell purities were assessed 24 h later using immunofluorescence with Tubulin β3 a neuronal marker and > = 90% of neuronal cells was obtained.

pEGFP-N1 plasmids were then used to transfect neuronal cells alone or co-transfected with indicated oligos using Lipofectamine2000 to improve visualization of neuron morphology [33]. Half the medium was replaced with fresh medium containing either sAPPα (10 nM) or *at*RA (2 µM) or vehicles before processed for further analysis. Z-stacked images acquired with Olympus FV3000 confocal microscope (at 1 μm step, 40 × objective) of at least 30 randomly chosen GFP-positive cells from each sample were used for Sholl analysis to calculate the number of dendrites intersections on concentric circles at 5 μm intervals using ImageJ [34].

### Cell lines and cultured cell transfections

The human embryonic kidney 293T cells, the mouse hippocampal neuronal cell line HT22 and mouse microglial cell BV2 were purchased from ATCC (Manassas, Virginia, USA). 293T, HT22 and BV2 cells were cultured in the Dulbecco’s modified Eagles medium (DMEM) with 10% FBS, 100 U/mL penicillin and 100 µg/mL streptomycin at 37℃ in a humidified atmosphere with 5% CO_2_. 293T and HT22 cells were plated on 6-well plates at the density of 4 × 10^5^/ well. The cells were transfected with 50 nM RNAs or 1 µg indicated plasmids using Lipofectamine 2000 transfection reagent according to the manufacturer’s instructions. The cell culture medium was replaced with fresh medium containing indicated treatments 24 h after transfection and maintained before processed to further analysis at indicated time points.

### ELISA

The amount of Aβ1–42 monomer in cells and tissues were determined using Amyloid beta 42 Mouse ELISA Kit (Cat. No. KMB3441, Invitrogen, Carlsbad, CA), according to the manufacturer’s instructions. The hemisphere hippocampal tissues were lysed in RIPA buffer (8 µL/ 1 mg of tissue) for 30 min on the ice, followed by centrifugation at 12,000 × *g* for 30 min. 5 µL supernatant of each sample was used for Aβ42 ELISA assay. The cells in 12-well plates were homogenized in RIPA buffer (60 µL per well) for 30 min on the ice. 50 µL supernatant of each sample was used for Aβ 42 ELISA assay.

### Golgi staining and dendrite arborization analysis

The FD Rapid Golgi Stain Kit (Cat. No. PK401, FD Neurotechnologies) was used for mouse brain to Golgi staining according to manufacturer’s instruction. Briefly, the mouse brain hemispheres (*n* = 4) obtained immediately after sacrifice were immersed into mercuric chloride solution (mixture of solutions A and B) for 2 weeks. The samples were then transferred to a cryoprotectant solution (solution C) and were kept in dark for another week. The samples were then frozen in isopentane on the dry ice and stored at -80℃. 100-µm thick serial coronal sections of hippocampus region were obtained using a cryostat microtome (CM1950, Leica). The slices were stained with ammonium hydroxide solution (mixture of solution D and E) for 10 min at room temperature, followed by 2 × brief wash with water. Slices were dehydrated with graded alcohols and cleared with xylenes, and then coverslipped using neutral balsam (BL704A, Biosharp, Hefei, China). Z-stack images (at 1 μm steps) of pyramidal neuron from hippocampus CA1 regions were captured under 20 × objective using EVOS FL Auto microscope (Thermo Fisher Scientific, Waltham, USA) and 3D images were reconstructed using Vaa3D software [35]. 10–15 pyramidal neurons per mouse were selected for Sholl analysis using ImageJ. The dendrite intersections on concentric circles with radius increased by 5 μm each starting from the cell soma were quantified.

### Phagocytosis assay of FITC-conjugated Aβ1–42

FITC-conjugated Aβ1–42 peptide (Cat. No. M212900, MREDA, Beijing, China) was dissolved in DMSO to make the 200 µM stock solution and stored at − 80 °C before use. 1 µM FITC-Aβ42 working solution was made 3 days before use by adding DMEM to the stock solution and incubation at 37 °C to allow for fibrillar Aβ aggregation. HT22 cells were transfected with indicated oligos or constructs for 12 h, followed by co-culturing with BV2 cells for 12 h. Mock-transfected cells were used as controls. Fibrillar Aβ solution was added to the co-cultured cells to a final concentration of 500 nM and incubated for 2 h to allow for Aβ phagocytosis. The cells were then fixed with 4% PFA at room temperature for 15 min and permeabilized with 0.2% Triton X-100 in PBS for 10 min with brief washes before and after. After blocking with 10% goat serum and 2% BSA in PBST for 1 h at room temperature, the cells were probed with anti-Iba1 (1:500, Cat. No. 019-19741, Wako, Japan) overnight at 4 °C. After washing with PBST three times, the cells were then incubated with Alexa Fluor 647 conjugate secondary antibody (1:800, Cat. No. 111-606-004, Jackson ImmunoResearch) for 1 h at room temperature. Cell nuclei were stained with Hoechst 33342 (1 µg/mL, Cat. No. B2261, Sigma-Aldrich) at room temperature for 10 min. The z-stack confocal images were captured at 0.7 μm step using 100× oil immersion objective and the 3D representative images were created using ImageJ’s 3D Viewer plugin. Nine optical fields were randomly chosen for each experiment and the z-stack images were captured under 40 × objective at 1 μm step size using Olympus FV3000 confocal microscope (Olympus, FV3000, Tokyo, Japan). The images were processed with FV31S-SW Viewer software (version 2.5) before quantification analysis of Aβ phagocytosis. The online software cellpose (version 2.0) was used for cellular segmentation, and ImageJ was used to assess the number of Aβ^+^ microglia and analysis the mean fluorescence intensity of FITC-Aβ1–42.

### Statistical analysis

All statistical analyses were performed using GraphPad Prism 8.0 software (GraphPad Software Inc., San Diego, CA, USA). Comparisons between two groups were performed using a two-tailed unpaired *Student’s t*-test. Multiple comparisons were performed using a one-way ANOVA followed by *post hoc* test. Data are expressed as mean ± SD or mean ± SEM, as indicated in the relevant figure legends. A probability of *p* < 0.05 was considered statistically significant.

## Results

### Identification and validation of the differentially expressed miRNAs associated with AD in blood plasma

The global expression profiles of miRNA in blood plasma samples taken from 11 AD patients as well as from 14 age and sex matched cognitively normal volunteers were analyzed using miRNA-seq. After the initial filtering, 1010 known miRNAs were successfully mapped using the reference miRBase (version 20.0) (Table S1). 108 miRNAs were concurrently expressed in both AD patients and controls, with one miRNA, *miR-4732-5p*, was detected only in the AD plasma (7 patients). The divergence in gene expression between AD patients and healthy control was demonstrated by principal component analysis (PCA) with normalized gene expression data (Fig. 1A). Total of 41 (24 upregulated and 17 downregulated) differentially expressed miRNAs (DEMs) with statistical significances were identified using two-tailed, unpaired *Student’s t*-test (adj. *p* < 0.05, Fig. 1B, Table S2). The differences in miRNA profiles of blood plasma from AD patients and controls were also demonstrated by the agglomerative hierarchical clustering of the DEMs using *Pearson* correlation coefficient and average distance, showing two main groups identified (*p* < 0.05, Fig. 1B). Among them, 29 DEMs (16 upregulated and 13 downregulated) has a |log2-fold change| >1 (FDR adjusted *p* < 0.05) (Fig. 1C). In addition, the expression of 7 miRNAs (*hsa-miR-425-3p*, *hsa-miR-192-5p*, *hsa-miR-1306-5p*, *hsa-miR-1307-3p*, *hsa-miR-150-5p*, *hsa-miR-423-3p*, *hsa-miR-4433b-5p*) in the blood plasma samples of all 12 AD patients was out of the 95% confidence intervals of their levels in the healthy controls (Table S3). These miRNAs, along with *miR-4732-5p* that was found to be exclusively expressed in the blood plasma of the AD patients, might be used as potential biomarkers for AD diagnosis and need to be verified. Next, the targets of these potential miRNAs which were related to cognitive dysfunction genes were selected (Fig. 1D). The result shown that two upregulated DEMs, *miR-140* and *miR-122*, which were conserved between human and mouse, came to our notion due to their potential functional significance in the dimension of the fundamental AD pathology. They both target ADAM10, the major α-secretase in CNS that is involved in APP processing (Fig. 1D). Also, terms of neuronal function, cell death and homeostasis, etc. were enriched in miRNA targets, related to cognitive dysfunction genes (Fig. 1E). The reproducibility of the expression of these two miRNAs was further verified in the blood plasma of an independent test cohort of AD patients and controls, as well as in the brain parenchyma of 6-month-old APP/PS1 mice and C57BL/6J control mice. The results of quantitative RT-PCR demonstrated the significantly upregulated expression of *miR-140* and *miR-122* in the blood plasma of AD patients, as well as in the hippocampi and cortex of APP/PS1 mice relative to the controls (Fig. 1E and G). We proposed that *miR-140* and *miR-122* might play critical roles in the pathological progression of AD and the potential mechanisms were further investigated in vitro and in vivo using APP/PS1 mouse model.

**Fig. 3:**
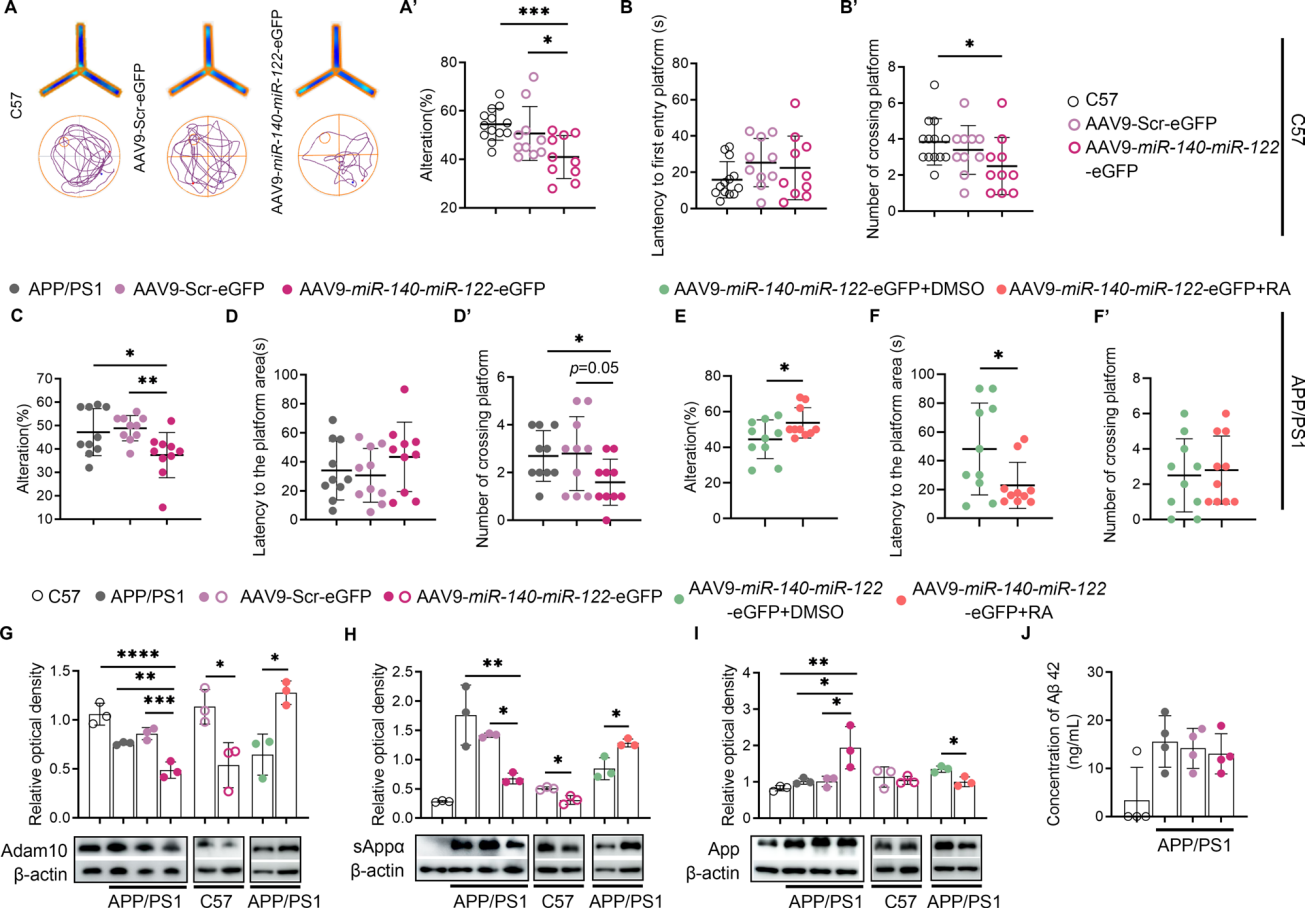
Overexpression of *miR-140* and *miR-122* altered proteolytic processing of APP and induced cognitive impairment in mice. (**A**-**B**). Behavioral assessment of C57BL/6J mice two months after intracerebroventricular injection of recombinant AAV9 vectors expressing *miR-140* and *miR-122* or scramble RNA, showing heatmap of animal’s position in Y maze and trajectory in Morris water maze (A), percentage of alternations in Y maze test (A’), latency to the first entry of platform (B) and number of crossings (B’) in Morris water maze. The results were presented as means ± SD (*n* = 13 for C57 mice; *n* = 10 for AAV9-Scr-eGFP; *n* = 10 for AAV9-*miR-140*-*miR-122*-eGFP). *p < 0.05, ***p < 0.001. Statistical analyses were performed using two-tailed unpaired two-tailed unpaired *Student’s t*-test. (**C**-**D**). Behavioral assessment of APP/PS1 mice two months after intracerebroventricular injection of recombinant AAV9 vectors expressing *miR-140* and *miR-122* or scramble RNA, showing percentage of alternations in Y maze test (**C**), latency to the first entry of platform (D) and number of crossings (D’) in Morris water maze. The results were presented as means ± SD (*n* = 10 for each group). *p < 0.05, **p < 0.01. Statistical analyses were performed using two-tailed unpaired *Student’s t*-test. (**E**-**F**). Behavioral assessment of APP/PS1 mice two months after injected with AAV9-*miR-140*-*miR-122*-eGFP with or without *at*RA treatment, showing percentage of alternations in Y maze test (E), latency to the first entry of platform (F) and number of crossings (F’) in Morris water maze. The results were presented as means ± SD (*n* = 10 for each group). *p < 0.05. Statistical analyses were performed using two-tailed unpaired *Student’s t*-test. (**G**-**I**). Results of western blot and densitometric quantification of the relative expression of ADAM10 (G), sAPPα (H) and APP (I) in the hippocampi of control C57 and APP/PS1 mice. C57 and APP/PS1 mice transfected with recombinant AAV9 vector expressing scramble RNA or *miR-140* and *miR-122*. APP/PS1 mice transfected with AAV9-*miR-140*-*miR-122*-eGFP were divided into two groups, which received *at*RA treatment or mock treatment, respectively. The expression of β-actin was used as an internal control. The results were presented as means ± SD (*n* = 3 for each group). *p < 0.05, **p < 0.01, ***p < 0.001, ****p < 0.0001. Statistical analyses were performed using One-way ANOVA or two-tailed unpaired *Student’s t*-test. (**J**). ELISA analysis of the level of Aβ42 in the hippocampi of the uninfected APP/PS1 mice as well as APP/PS1 mice transfected with recombinant AAV9 vector expressing scramble RNA or *miR-140* and *miR-122*. The uninfected C57 mice was used as the controls

### Overexpression of *miR-140* and *miR-122* reduced non-amyloidogenic processing of APP via targeting ADAM10

*miR-140* and *miR-122* mimics were transfected either individually or in combination into mouse hippocampal neuron cells (HT22) as well as into HEK-293T cells. Knock-down of the endogenous expression of *miR-140* and *miR-122* in these cells were accomplished by transfection of recombinant pcDNA3.1 plasmids overexpressing the specific inhibitory sequences of *miR-140* and *miR-122* “Tough Decoy” (TuD) that contained two copies of miRNA-binding sites of each miRNA (Fig. 2A and B). Transfection of pcDNA3.1-*miR-140*-*miR-122*-TuD significantly reduced the endogenous expression of *miR-140* and *miR-122* in cultured 293T cells (Fig. 2C). Compared with that of the mock-transfected control cells or the control cells transfected with scramble RNA, overexpression of *miR-140* or *miR-122* significantly down-regulated the protein expression of ADAM10 (Fig. 2D and G). Co-expression of *miR-140* and *miR-122* mimics further inhibited the expression of ADAM10 in both 293T and HT22 cells (Fig. 2D and G). On the contrary, inhibition of the endogenous *miR-140* and *miR-122* expression in these cells by overexpressing TuD containing tandem inhibitors of *miR-140* and *miR-122* resulted in up-regulated ADAM10 (Fig. 2D and G). In accordance, accumulated APP and decreased soluble APPα were demonstrated in the cells overexpressing *miR-140* and *miR-122* and vice versa (Fig. 2E, F, H and I). The imbalance between amyloidogenic and non-amyloidogenic processing of APP in cells overexpressing *miR-140* and *miR-122* was likely to be ascribed to the reduced ADAM10 expression, since administration of retinoic acid, an ADAM10 promoter inducer, restored the expression of ADAM10 as well as the α-proteolysis of APP in *miR-140*/*miR-122* overexpressing cells. Although significant changes of expression were not observed, nonsignificant trend of the expression changes in APP and sAPPα were shown in HT22 cells overexpressing either *miR-140* or *miR-122* in our results of western blot assay, indicating a cumulative effect of *miR-140* and *miR-122* via targeting the common downstream target ADAM10 (Fig. 2H and I). Although no significant influence on Aβ production was observed in HT22 cells with *miR-140*/*miR-122* overload, depletion of endogenous *miR-140* and *miR-122* by overexpressing *miR-140*-*miR-122*-TuD significantly reduced Aβ production in HT22, indicating a redundancy in APP processing and the therapeutic potentials of these miRNAs in AD treatment (Fig. 2J).

### Co-overexpression of *miR-140* and *miR-122* induced memory impairments in wild type mice and aggravated cognitive dysfunction in APP/PS1 mice

Memory impairments along with other AD pathologies including Aβ deposition, abnormal long-term potentiation appears between 3–6 months in APP/PS1 mice [36]. Recombinant AAV9 virus overexpressing both *miR-140* and *miR-122* (AAV9-*miR140*-*miR-122*-eGFP) were constructed for in vivo functional assays. A total number of 8 × 10^10^ genome copies AAV9-*miR-140*-*miR-122*-eGFP or control virus AAV9-Scr-eGFP were injected into both sides of the lateral ventricles in 4-month-old APP/PS1 mice as well as in 4-month-old wild type C57BL/6J mice to induce overexpression of *miR-140* and *miR-122* in widespread regions in mouse brains. Age-matched untreated mice were used as controls. Expression and region of infection of the recombinant virus in mouse brains were verified at two months after the injection (Fig. S1). Behavioral test batteries comprised of the Y maze and Morris water maze were performed two month after the injection to assess the short-term working memory and the spatial learning, respectively. Overexpression of *miR-140* and *miR-122* induced impaired memory in wild type mice and aggravated cognitive deterioration in AD mice, demonstrated by the reduced alternations in the Y maze and reduced number of platform-crossing in the probe trials in water maze, respectively, compared with their counterparts injected with AAV9-Scr-eGFP as well as the controls (Fig. 3A-3D, 3A’-3D’). Administration of *all-trans* retinoic acid (20 mg/g), which was shown to upregulate ADAM10 transcription via binding to the two retinoid X-receptor/retinoic acid receptor (RXR/RAR) in the promoter, significantly increased the alternations in Y maze as well as decreased the escape latencies in the Morris water maze in the AD mice injected with AAV9-*miR140*-*miR-122*-eGFP, comparing with the AD mice received vehicle treatment (Fig. 3E, F and F’). The expression of ADAM10, sAPPα, APP and Aβ in the hippocampi of these mice were evaluated using western blot and ELISA assay, respectively. Compared to their counterparts injected with AAV9-Src-eGFP, the expression of ADAM10 and sAPPα was significantly reduced in the hippocampi of APP/PS1 and C57 mice injected with AAV9-*miR140*-*miR-122*-eGFP while the levels of APP were significantly increased only in the hippocampi of APP/PS1 mice overexpressing *miR-140* and *miR-122* (Fig. 3G-I). The mice infected with the control AAV9-Scr-eGFP virus displayed no significant change of the levels of these proteins, comparing with those of the uninfected controls. Administration of *at*RA restored the expression of ADAM10 and sAPPα while reduced APP in the brain of APP/PS1 mice injected with AAV9-*miR-140*-*miR-122*-eGFP (Fig. 3G-I). However, in line with what observed in in vitro analysis using cultured cells, the results of ELISA assay showed no significant change in the levels of Aβ monomers in the hippocampi of APP/PS1 mice transfected with either AAV-Src-eGFP or AAV-*miR-140*-*miR-122*-eGFP (Fig. 3J). These observations indicated the detrimental effects of increased *miR-140* and *miR-122* expression in AD pathology via targeting ADAM10 were unlikely to be ascribed to enhanced complimentary β-cleavage of APP.

### Dysregulated *miR-140* and *miR-122* expression affected microglial chemotaxis and Aβ phagocytosis

**Fig. 4:**
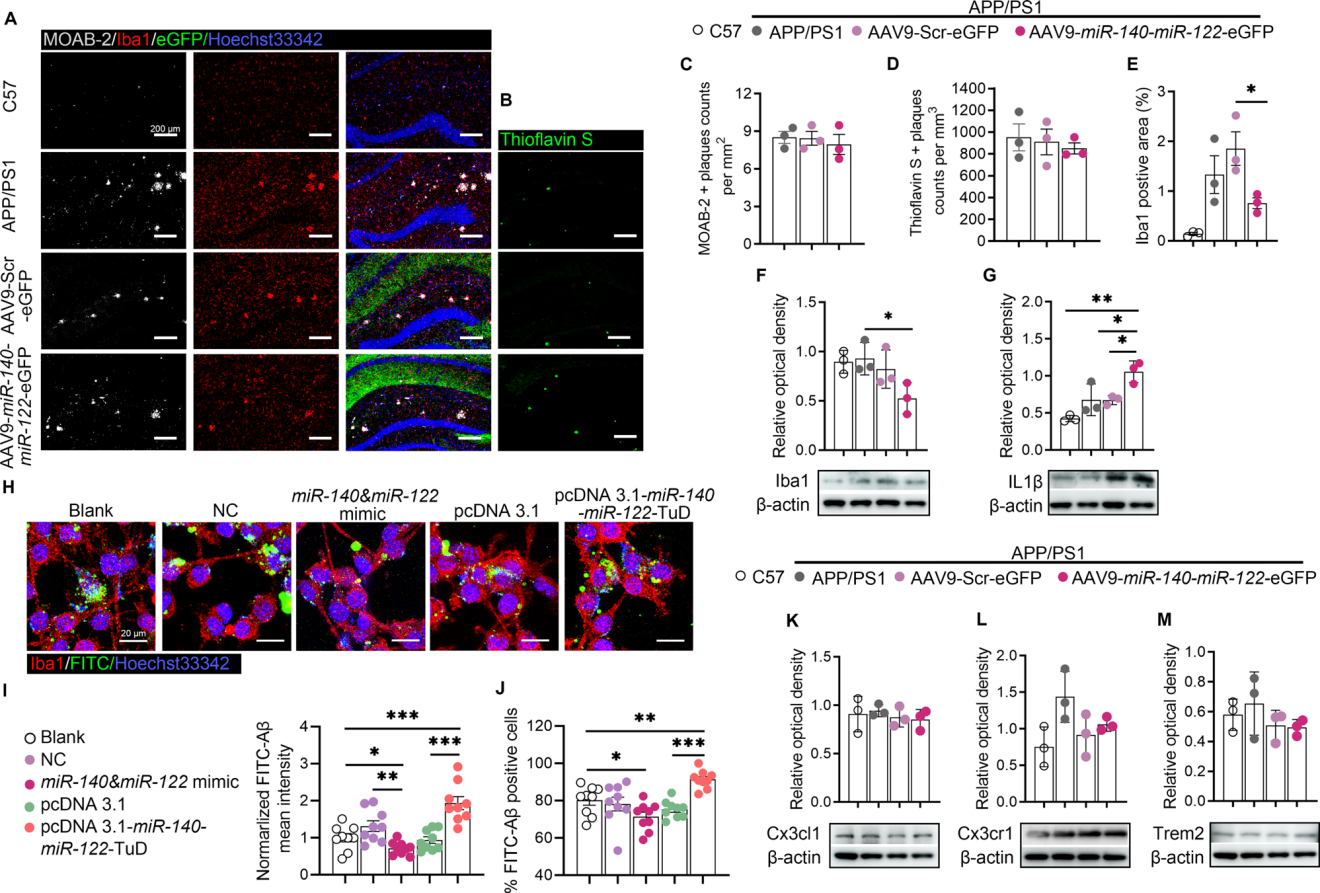
Overexpression of *miR-140* and *miR-122* reduced microgliosis and microglia phagocytosis. (**A**-**B**). Representative confocal z-stack images showing results of immunohistochemical analysis in the DG subregions on coronal sections of the hippocampus of control C57 and APP/PS1 mice, which were transfected with AAV9-Scr-eGFP or AAV9-*miR-140*-*miR-122*-eGFP, respectively. Aβ deposition was stained with MOAB-2 (white) or ThioS (green). Microglia cells were stained using anti-Iba1 (red). Scale bar: 200 μm. (**C**-**E**). Quantitative analysis of the immunofluorescence intensities of three consecutive sections of three independent biological replicates, showing average number of MOAB-2^+^ Aβ plaques (**C**), ThioS^+^ fibrilar Aβ plaques (**D**) and percentage of Iba1 postive area (**E**). Results were presented as meam ± SEM. *p < 0.05. Statistical analyses were performed using One-way ANOVA. (**F**-**G**). Results of western blot analysis and densitometric quantifications of the relative expression of Iba1 (**F**) and IL-1β (**G**) in the hippocampi of APP/PS1 mice transfected with AAV9-Scr-eGFP or AAV9-*miR-140*-*miR-122*-eGFP, respectively. Untransfected APP/PS1 mice and wild type C57 mice were used as controls. The expression of β-actin was used as an internal control. The results were presented as means ± SD (*n* = 3). *p < 0.05, **p < 0.01. Statistical analyses were performed using One-way ANOVA. (**H**-**J**). Aβ phagocytosis analysis in BV2 cells co-cultured with untransfected HT22 cells or HT22 cells that were transfected with scramble miRNAs, mimics of *miR-140* and *miR-122*, pcDNA3.1, or pcDNA3.1-*miR-140*-*miR-122*-TuD, respectively. Representative images of immunofluorescence showing Iba1 (red) labelled BV2 cells and FITC-Aβ (green) (**H**). Quantitative analysis of fluorescence intensities showing the average fluorescence intensity of FITC-Aβ in phagocytic BV2 cell (**I**) and proportion of Aβ phagocytic BV2 cells (**J**). The results were presented as means ± SEM (*n* = 9). *p < 0.05, **p < 0.01, ***p < 0.001. Statistical analyses were performed using One-way ANOVA. Scale bar: 20 μm. (**K**-**M**). Western blot analysis and densitometric quantification of the relative expression of Cx3cl1 (**K**), Cx3cr1 (**L**) and Trem2 (**M**) in the hippocampi of APP/PS1 mice transfected with AAV9-Scr-eGFP or AAV9-*miR-140*-*miR-122*-eGFP, respectively. Untransfected APP/PS1 mice and wild type C57 mice were used as controls. The expression of β-actin was used as an internal control. The results were presented as means ± SD (*n* = 3). Statistical analyses were performed using One-way ANOVA

Moreover, the potential influence of dysregulated *miR-140* and *miR-122* expression on APP metabolism and Aβ deposition was further investigated in mice injected with AAV9-*miR-140*-*miR-122*-eGFP using immunohistochemical analysis with monoclonal anti-Aβ antibodies (MOAB-2) as well as using Thioflavine S staining, respectively. As expected, amyloid plaques were observed in APP/PS1 mice while were negative in wild type mice (Fig. 4A). Compared with their untransfected counterparts or the controls transfected with AAV9-Src-eGFP, overexpression of *miR-140* and *miR-122* did not significantly promote amyloid plaque deposition in either APP/PS1 mice or wild type mice with stereotactic injection of AAV9-*miR140*-*miR-122*-eGFP, shown by both MOAB-2 and Thioflavine S (ThioS) staining (Fig. 4A-D). In accordance with the increased Aβ deposition in APP/PS1 mice, significantly enhanced microglial activities were demonstrated by the increased immunofluorescence intensities of Iba1 observed in the hippocampi of APP/PS1 mice as well as in the hippocampi of APP/PS1 mice transfected with AAV9-Scr-eGFP, compared with the control C57 mice (Fig. 4A and E). On the contrary, instead of triggering microgliosis, the results of immunohistofluorescence showed significantly reduced Iba1 immunoreactivities in the hippocampi of APP/PS1 mice overexpressing *miR-140* and *miR-122* (Fig. 4A and E). Results of western blot analysis confirmed the reduction of Iba1 expression in the hippocampi of mice overexpressing *miR-140* and *miR-122*, although the expression of proinflammatory cytokines IL-1β was dramatically increased (Fig. 4F and G). These results led to our speculation of defective hippocampal microglia reactivity in the brains of the mice overexpressing *miR-140* and *miR-122*, where the aggravated neuroinflammation was observed comparing with that of the control mice. This hypothesis was further supported by Aβ phagocytosis analysis in vitro. Mimics of *miR-140* and *miR-122* and pcDNA3.1-*miR-140*-*miR122*-TuD were overexpressed in cultured HT22 cells, respectively. Untransfected cells, cells transfected with scramble miRNAs or pcDNA3.1, were used as controls. These cells were then co-cultured with BV2 cells and the phagocytotic activities of FITC-labeled Aβ42 were quantified. Our results showed that overexpressing *miR-140* and *miR-122* in HT22 significantly suppressed the phagocytotic activities in the co-cultured BV2 cells while downregulation of *miR-140*/*miR-122* expression in HT22 significantly increased the number of phagocytotic cells as well as phagocytotic activities in the co-cultured BV2 cells (Fig. 4H-J). These results were in correlation with our previous observation of the reduced microglial activities and the increased sizes of Aβ plaques in the hippocampi of mice overexpressing *miR-140* and *miR-122*.

Given that both Fractalkine (CX3CL1) and TREM2, a highly expressed chemokine on neuronal cells that mediate neuron-to-glia communications via its receptor (CX3CR1) on microglia and a single-transmembrane domain protein expressed on microglia that function as a sensor of apolipoproteins in Aβ plaques, were also targeted by ADAM10’s proteolytic cleavage, the expression of CX3CL1/CX3CR1 and TREM2 was further evaluated in our mouse models [37]. No significant changes in these proteins’ expression in the hippocampi of APP/PS1 mice overexpressing *miR-140*/*miR-122* were revealed by the western blot assay, compared with that of the mice overexpressing scramble RNA and that of the uninfected control mice (Fig. 4K and M). Collectively, the results suggested that the restrained microglial chemotaxis/phagocytosis observed both in vivo and in vitro with upregulated *miR-140* and *miR-122* expression was independent of dysregulation of these receptors.

### The reduced neuroprotective plaque-associated microglia exacerbated neurite dystrophy around the amyloid plaque

**Fig. 5:**
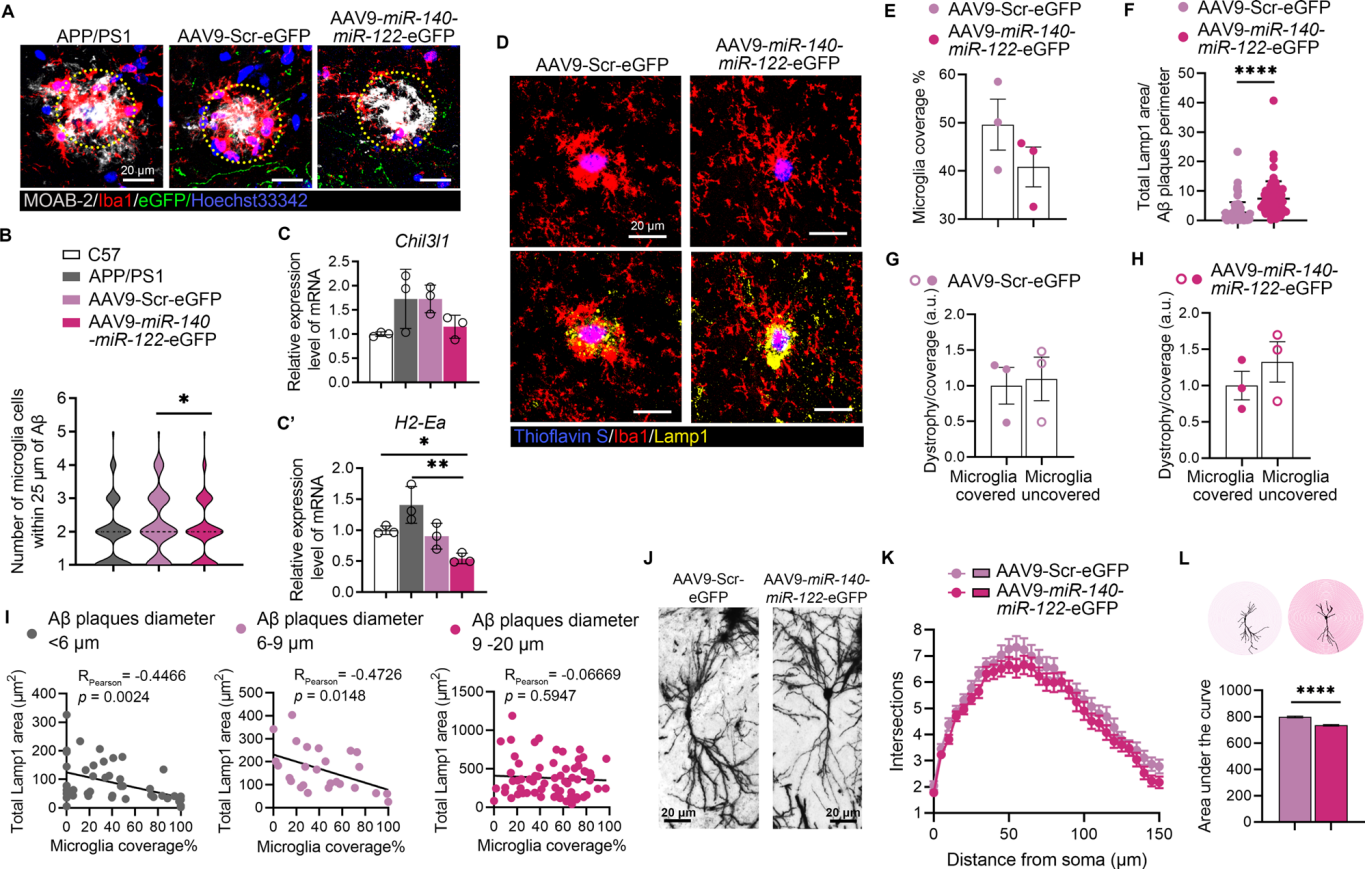
Overexpression of *miR-140* and *miR-122* reduced microglia reactivity and increased Aβ plaque neurotoxicity. (**A**). Representative confocal z-stack images of immunohistochemical analysis, showing Aβ plaques (MOAB-2, white) and plaque-associated microglia (Iba1, red) in the DG region of untransfected APP/PS1 mice, APP/PS1 mice transfected with AAV9-Scr-eGFP or AAV9-*miR-140*-*miR-122*-eGFP, respectively. Nuclei were counterstained with Hoechst33342 (blue). Scale bar: 20 µm. (**B**). Quantification of the number of plaque-associated microglia within 25 µm diameter from the center of Aβ plaques in the DG subregions on coronal sections of the hippocampi of APP/PS1 mice, APP/PS1 mice transfected with AAV9-Scr-eGFP or AAV9-*miR-140*-*miR-122*-eGFP, respectively. Results were recorded on three consecutive sections of three independent biological replicates. *n* > 150 plaques for each group. The median of each group was indicated with the black dash lines. *p < 0.05. Statistical analyses were performed using One-way ANOVA. (**C**-**C’**). Results of quantitative RT-PCR showing the relative expression of *Chi3l1* and *H2-Ea* in the hippocampi of C57BL/6J mice, the APP/PS1 mice, APP/PS1 mice transfected with AAV9-Scr-eGFP and APP/PS1 mice transfected with AAV9-*miR-140*-*miR-122*-eGFP, respectively. The results were presented as means ± SD (*n* = 3). **p < 0.01. Statistical analyses were performed using One-way ANOVA. (**D**). Representative confocal z-stack images of immunohistochemical analysis of microglia coverage and neurite dystrophy, showing ThioS^+^ Aβ plaques (blue), microglia (Iba1, red), and dystrophic neurite (Lamp1, yellow). Scale bar: 20 μm. (**E**-**H**). Quantification of the microglia coverage over the plaques (**E**) and Lamp1 area (**F**) around each individual plaque. Quantification of the area of dystrophic neurites extending radially outward from microglia-covered or -uncovered plaque perimeter in the hippocampi of APP/PS1 mice transfected with AAV9-Scr-eGFP (**G**) or AAV9-*miR-140*-*miR-122*-eGFP (**H**). Results of three consecutive sections of three independent biological replicates were presented as means ± SEM or means ± SD (*n* > 70 plaques for each group). ****p < 0.0001. Statistical analyses were performed using two-tailed unpaired *Student’s t*-test. (**I**). Correlation analysis of the total Lamp1 positive area and microglia coverage for each individual plaques, which were grouped into 3 different ranges of sizes plaques. Over 70 plaques were selected from APP/PS1 mice transfected with AAV9-Scr-eGFP (*n* = 3) or AAV9-*miR-140*-*miR-122*-eGFP (*n* = 3), respectively. (**J**). Golgi-Cox staining showing pyramidal neurons in the hippocampal CA1 of APP/PS1 mice transfected with AAV9-Scr-eGFP or AAV9-*miR-140*-*miR-122*-eGFP, respectively. Scale bar: 20 μm. (**K**-**L**). Sholl analysis of the dendric intersections of pyramidal neurons. The number of basal dendritic intersections (**K**) and the area under the curve (**L**) were quantified. Results are expressed as means ± SD. ****p < 0.0001. Statistical analyses were performed using two-tailed unpaired *Student’s t*-test

Given that excessive Aβ cause neuroinflammation and activate microglia/astroglia to carry out phagocytosis of Aβ plaques [38], the numbers of microglia around Aβ deposition (immuno-stained with MOAB-2) were calculated within 25 µm diameter from the center of Aβ plaques. Significantly reduced numbers of microglia around Aβ plaques were identified in the dentate gyrus of AAV9-*miR140*-*miR-122*-eGFP infected APP/PS1 mice, compared to their counterparts infected with AAV9-Scr-eGFP, indicating the decreased microglial migration toward Aβ plaques (Fig. 5A and B). The reduced microglial migration in the hippocampi of mice overexpressing *miR-140* and *miR-122* was in line with the downregulation of reactive microglia signature, including *Chi3l1* and *H2-Ea*, as shown by the quantitative real-time PCR analysis (Fig. 5C and C’). 16–40 ThioS labeled hippocampal Aβ fibrils per mouse were subsequently selected. The microglia coverage over the plaques were subsequently quantified. Compared with the mice infected with AAV9-Scr-eGFP, the overall microglial enveloping of plaques was significantly reduced in the hippocampi of APP/PS1 mice overexpressing *miR-140*/*miR-122* (Fig. 5D and E). Given the neurotoxicity around Aβ aggregates, we further assessed the neuritic dystrophy within the plaque regions in the mouse brains with or without *miR-140*/*miR-122* overexpression. Compared with the APP/PS1 mice infected with AAV9-Scr-eGFP, quantification of the z-stack images of immunolabelled dystrophic neurites using anti-Lamp1 revealed significantly escalated areas of swollen dystrophic neutites in the juxta-Aβ plaque regions in mice overexpressing *miR-140* and *miR-122* (Fig. 5D and F). The neurotoxicity was significantly exacerbated in the sectors outwards microglia-uncovered fractions in the APP/PS1 mice overexpressing *miR-140*/*miR-122* as well as in the control APP/PS1 mice overexpressing scramble RNA (Fig. 5G and H). Significant negative correlations between the areas of neuritic dystrophy and the microglia coverage were noticed, especially around the amyloid plaques with diameter smaller than 9 μm (Fig. 5I). The results indicated the weakened neuroprotection of microglia barrier within the plaque-adjacent microenvironment in the brains of the mice with dysregulated *miR-140* and *miR-122* expression. Further assessment of dendrite arborization of well-stained pyramidal neurons in the ipsilateral hippocampal CA1 regions was performed using Sholl analysis (Fig. 5J). Compared with that of the mice injected with AAV9-Scr-eGFP, quantification of basal dendritic intersections on concentric circles drawn from the cell body at 5 μm intervals revealed reduced dendritic extension in the hippocampi of mice overexpressing *miR-140* and *miR-122*, indicating the exacerbated neuronal hypotrophy (Fig. 5K and L).

### The neurotoxicity of *miR-140* and *miR-122* overexpression was alleviated by additional sAPPα

**Fig. 6:**
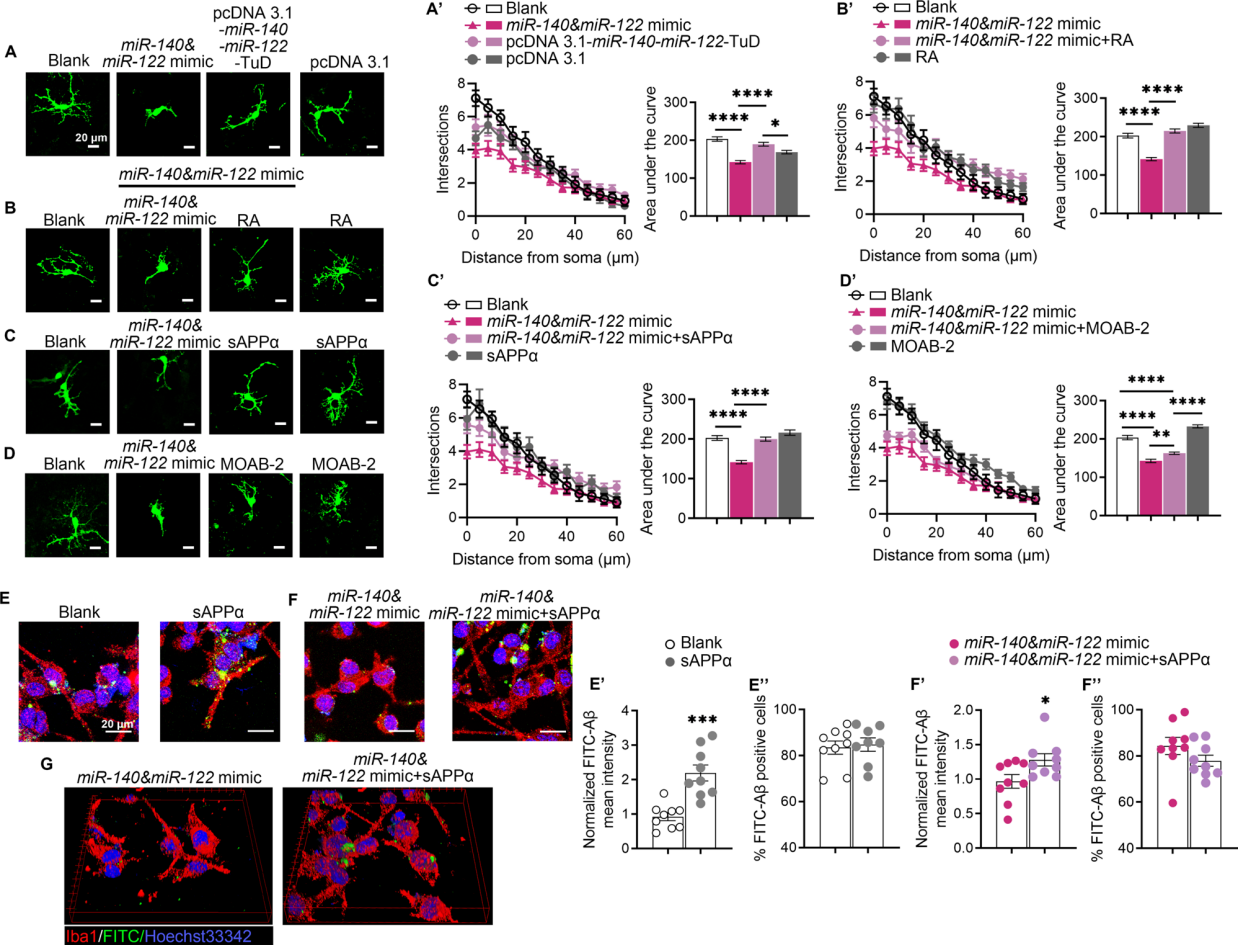
Neurite outgrowth and microglia phagocytosis impaired by *miR-140* and *miR-122* overexpression was restored by administration of sAPPα in vitro. (**A**-**D**). Representative fluorescence images of primary cortical neuron cells transfected with mimics of *miR-140* and *miR-122*, pcDNA3.1, or pcDNA3.1-*miR-140*-*miR-122*-TuD (A), respectively, followed by treatment of *at*RA (**B**), sAPPα (**C**) and Aβ blockage (**D**). pEGFP was co-transfected for visualization. Cells transfected with GFP constructs and followed by mock treatment were used as controls. (A’-D’). Quantification of the dendric intersections and area under the curve using Sholl analysis. The results were presented as means ± SEM (*n* > 30 neurons for each group). *p < 0.05, **p < 0.01, ****p < 0.0001. Statistical analyses were performed using One-way ANOVA. (**E**-**E**’’). Analysis of Aβ phagocytosis in BV2 cells co-cultured with HT22 cells that were transfected with scramble miRNAs with or without administration of 10 nM sAPPα. Representative immunofluorescence images (**E**) of microglia Aβ phagocytosis, showing BV2 cells (anti-Iba1, red) and FITC-Aβ (green). Quantification of Aβ phagocytosis, showing internalized fluorescence intensity of FITC-Aβ per BV2 cell (E’) and the proportion of Aβ phagocytic BV2 cells (**E**’’). (**F**-**F**’’). Analysis of Aβ phagocytosis in BV2 cells co-cultured with HT22 cells that were transfected with mimics of *miR-140* and *miR-122* with or without administration of 10 nM sAPPα. Representative immunofluorescence images (**F**) of microglia Aβ phagocytosis, showing BV2 cells (anti-Iba1, red) and FITC-Aβ (green). Quantification of Aβ phagocytosis, showing internalized fluorescence intensity of FITC-Aβ per BV2 cell (**F**’) and the proportion of Aβ phagocytic BV2 cells (**F**’’). (**G**). 3D representations of confocal z-stack images of microglia Aβ phagocytosis. The results were presented as means ± SEM (*n* = 9). Scale bar: 20 μm. *p < 0.05, ***p < 0.001. Statistical analyses were performed using two-tailed unpaired *Student’s t*-test

Given that overexpression of *miR-140*/*miR-122* significantly reduced α-proteolysis of APP both in vitro and in vivo without significant influence on Aβ deposition, we further asked whether the neuritic dystrophy and dysregulated microglial chemotaxis induced by excessive *miR-140*/*miR-122* were due to mitigated sAPPα protection. The primary cortical neuron cells were prepared from E15.5 mouse embryos. The primary neurons were transfected with *miR-140* and *miR-122* mimics, followed by treatment with recombinant sAPPα, anti-Aβ42 blockade or *at*RA, respectively. The control cells were transfected with scramble RNA, followed by mock treatments. GFP-positive cells were randomly selected for dendrite arborization analysis. Neurons transfected with mimics of *miR-140* and *miR-122* showed diminution of dendritic length and reduced in dendritic complexity compared with that of the neurons transfected with scramble RNA (Fig. 6A-6A’’). However, compared with the cells transfected with the expression vectors, increased dendritic length and complexity were observed in neuronal cells with downregulated endogenous *miR-140* and *miR-122* by overexpressing *miR-140*-*miR-122*-TuD (Fig. 6A and A’’). The reduced number of dendritic branches and the outgrowth of total neurite in neurons transfected with mimics of *miR-140* and *miR-122* were restored by addition of sAPPα and *at*RA, respectively, while depletion of Aβ with anti-Aβ blockade showed minimal improvements in dendrite arborization in these cells (Fig. 6B and B’’, 6 C–6 C’’, 6D-6D’’). These results indicated that the adverse effects of overexpression of *miR-140* and *miR-122* were likely to be ascribed to the reduced production of sAPPα due to downregulation of ADAM10.

Given the reduced microglia migration and coverage over Aβ plaque observed in the hippocampi of mice overexpressing *miR-140*/*miR-122* as well as the reduced Aβ phagocytosis in BV2 cells co-cultured with neuronal cells overexpressing *miR-140*/*miR-122*, the potential beneficiary effects of sAPPα on microglia phagocytosis was evaluated in vitro. We found that additional sAPPα significantly increased the phagocytic activities of FITC-labeled Aβ42 in BV2 cells, compared with that of the mock-treated control cells (Fig. 6E-6E’’). In addition, administration of sAPPα also restored the impaired phagocytotic capacities the microglial cells co-cultured with HT22 cells transfected with *miR-140* and *miR-122* mimics, although the number of phagocytotic cells was not affected (Fig. 6F and F’’, 6G). The results collectively suggested that the reduced production of neuroprotective sAPPα was a key player in the elevated Aβ neurotoxicity and exacerbated the progression of AD-like pathology in mice with dysregulated *miR-140* and *miR-122* expression.

## Discussion

Human brain expresses more than 70% of microRNAs, aberrant expression of which have been shown to associate with the pathological progression of AD by modulating amyloidogenesis and Tau pathology, as well as unknown molecular pathways [39]. The differently expressed microRNAs may be reflected in the profiles of circulating small non-coding RNAs and serve as prognostic biomarkers for AD diagnosis at early stages [40]. For instance, *miR-206*, targeting brain-derived neurotrophic factor (BDNF) and *miR-29*, targeting β-site amyloid precursor protein cleaving enzyme 1 (BACE1), were among the most addressed microRNA signatures of AD [41, 42]. In this study, the levels of 16 miRNAs were found significantly increased in the blood plasma of AD patients. Among them, the plasma levels of 7 miRNAs, were outside of the 95% confidence intervals of their healthy controls, along with *miR-4732-5p* that exclusively detected in AD plasma, formed an 8-member group of miRNA signature that might be used for AD biomarker candidates. Although not consistently changed in all plasma samples of AD patients, the two significantly upregulated miRNAs, *miR-140* and *miR-122*, both targeting ADAM10, the major form of α-secretase in brain, came to our notion due to the mechanistic dimensions. Overexpression of *miR-140* and *miR-122* in CNS induced cognitive impairment in wild-type mice and exacerbated cognitive dysfunction in APP/PS1 mice. Although neither significant changes in the number and size of amyloid plaques nor significantly increased levels of overall Aβ42 were observed in these mice, compared to their untransfected counterparts and counterparts transfected with control virus, significantly decreased sAPPα production associated with *miR-140*/*miR-122* overexpression and thus downregulated ADAM10 were verified both in vitro and in vivo. These results indicated that although the ADAM10 mediated α-proteolysis of APP was likely to be the primary source of sAPPα production, alternative pathways, including ubiquitylation and other α-/β-like cleavage may play redundant roles in APP metabolism [43]. In addition, given that the majority of APP is retained in Golgi and only 10% of APP eventually transported to the plasma membrane, it was not surprising that downregulation of ADAM10 by overexpressing *miR-140* and *miR-122* had minor influence in the level of total cellular APP [44].

There is ongoing controversy regarding the correlation between Aβ levels and cognitive function in the pathogenesis of AD [45]. Although Aβ buildup in brain was one of the earliest molecular pathological changes detected even decades before AD symptoms, the progression of Aβ deposition showed weak correlation, if any, with the severities of this condition [46]. The two most relevant forms of β-amyloid peptides are Aβ40 and Aβ42, which are more likely to form self-aggregates. Among numerous Aβ species, including Aβ monomer, oligomer, protofibrils and fibrils, Aβ oligomer and protofibrils are considered toxic while Aβ monomers have been shown to be neuroprotective [47]. Oligomeric Aβ, Aβ protofibrils and Aβ deposits activate microglia and induce inflammatory response via inflammasome signaling as well as chemokine receptors [48]. Excessive Aβ and tau also interfere with regulated autophagy, induce pro-apoptotic proteins, and eventually lead to neuronal death, which in turn, aggravate neuroinflammation by releasing damage-associated molecular patterns (DAMPs). On the other hand, microglia express various chemokine receptors that can be activated by Aβ to initiate chemotaxis, a cellular event that is important for microglia mediated Aβ clearance by phagocytosis and hydrolysis [49]. Nonetheless, uncontrolled microglia reaction can result in destructive neuroinflammation, which is one of the neuropathological hallmarks of AD [50]. Besides passive response induced by Aβ and damaged neurites, microglia cells were suggested to play causative roles in early AD pathogenesis. Many genes within the risk loci for AD, e.g. *TREM2* and *CD33*, have been found expressed actively in microglia and affect immunosurveillance, which are essential for maintaining CNS homeostasis [49]. On the contrary to the activated microgliosis around Aβ plaques that commonly seen in AD-related neuroinflammation, the number of microglia cells in DG as well as within the 25 μm radius plaque-juxtaposed area were decreased in mice overexpressing *miR-140* and *miR-122*. Further analysis revealed significant reduction of the microglia coverage over compact ThioS^+^ Aβ plaques in the mice overexpressing *miR-140*/*miR-122*, which was correlated with the significantly increased neurotoxicity and neurite dystrophy. Given that neuron-derived CX3CL1 is also cleaved by ADAM10 to release soluble form of CX3CL1, which serves as chemoattractant, we proposed that CX3CL1 was less cleaved in mice overexpressing *miR-140*/*miR-122* due to downregulated ADAM10 with resultant decrease in chemotactic CX3CL1 and increase in the membrane-bound suppressive CX3CL1. However, neither decreased soluble CX3CL1 nor increased static form of CX3CL1 was observed in the mice overexpressing *miR-140* and *miR-122* in the brain. Therefore, the proteolytic cleavage of this chemokine in the brains of the mice overexpressing *miR-140* and *miR-122* was likely to be maintained by functional redundancies of disintegrins and metalloproteinase including ADAM10 and ADAM17 [37]. Likewise, western blot analysis of the microglial specific immunoreceptor TREM2 (triggering receptor expressed on myeloid cell 2), which could also be cleaved by ADAM10, demonstrated no significant change regarding it’s cleavage in the brain tissues of the mice overexpressing *miR-140* and *miR-122*. Given that ADAM10 principally acts at the cell surface, downregulation of ADAM10 in neuronal cells may have limited impact on the cleavage of microglial TREM2 [51].

sAPPα, the non-amyloidogenic product of ADAM10, has been well characterized for its neuroprotective roles and its therapeutic potentials in Alzheimer’s disease [52]. It has been shown to protect neuronal survival in adverse conditions both in vitro and in vivo [16, 53]. Exogenous sAPPα or sAPPα overexpression have been shown to prevent AD pathology and improve learning and memories in AD mouse models [54–56]. sAPPα also displayed neurotrophic activities in promoting the proliferation and neurite outgrowth of neural progenitor cells (NPC), possibly via activating ERK and MAP-kinase signaling pathways [15, 57]. In this study, we found that transfection of mimics of *miR-140* and *miR-122* significantly reduced the mouse embryonic neural cells neurite outgrowth, which could be rescued by additional sAPPα but not by anti-Aβ antibody neutralization. Likewise, the attenuated microglial chemotaxis-phagocytosis in cultured BV2 cells transfected with mimics of *miR-140* and *miR-122*, including both the percentage of phagocytic microglia and the phagocytic index, was also restored by exogenous sAPPα. Previous research demonstrated that sAPPα interacted with microglial receptors, such as class A SR, LRP1 and Group II mGluR, to enhance Aβ phagocytosis [58, 59]. As a dominant strategy in AD intervention, the rationale underlying a causative role for Aβ in AD pathogenesis has been constantly challenged, despite of the recent approval of lecanemab in AD therapy [60].

## Conclusions

Two miRNAs, *miR-140* and *miR-122*, were found significantly increased in the peripheral blood samples of AD patients as well as in the brain tissues of APP/PS1 mice. Overexpression of *miR-140* and *miR-122* in the hippocampus induced cognitive impairment in wild type C57 mice and exacerbated cognitive deterioration in APP/PS1 mice. Given that ADAM10, the major α-secretase in CNS, is the common downstream target of *miR-140* and *miR-122*, the potential influence of dysregulated *miR-140*/*miR-122* expression on APP metabolism and AD-related pathologies have been studied in mice with ectopic hippocampal *miR-140* and *miR-122* expression as well as in vitro. Our results suggested that reduced α-proteolytic cleavage of APP and the consequential reduced production of sAPPα was likely to be the key player in mediating the pathological outcomes of dysregulated expression of *miR-140* and *miR-122* in CNS, by showing unaltered soluble Aβ levels and Aβ deposits in APP/PS1 mice overexpressing *miR-140*/*miR-122* and that administration of RA to induced ADAM10 expression significantly improved the performance of these mice in behavioral assessment. In addition, either overexpression of *miR-140*/*miR-122*-specific TuD or treatment of RA or sAPPα, but not Aβ depletion, could improve the suppressed neurite outgrowth of embryonic neuronal cells transfected with mimics of *miR-140* and *miR-122* as well as the reduced Aβ phagocytosis in co-cultured microglia. Our results support a promising strategy in AD therapy by manipulating multiple targets in the α-proteolytic pathway of APP.

### Electronic supplementary material

Below is the link to the electronic supplementary material.

Supplementary Material 1

Supplementary Material 2

Supplementary Material 3

Supplementary Material 4

Supplementary Material 5

## Data Availability

No datasets were generated or analysed during the current study.

## Abbreviations

AD: Alzheimer’s disease
Aβ: Accumulated beta-amyloid
APP: Amyloid precursor proteins
sAPPα: Soluble APPα
*at*RA: *All-trans* retinoic acid
MMSE: Mini-Mental State Examination
MWM: Morris water maze
TMM: Trimmed mean of M-values
TPM: Transcripts per million
GO: Gene Ontology
KEGG: Kyoto Encyclopedia of Genes and Genomes
PCA: Principal component analysis
DEMs: Differentially expressed miRNAs
BDNF: Brain-derived neurotrophic factor
BACE1: β-site amyloid precursor protein cleaving enzyme 1
DAMPs: Damage-associated molecular patterns
TREM2: Triggering receptor expressed on myeloid cell 2
NPC: Neural progenitor cells
