# Supplementary material for: Dysregulated expression of miR-140 and miR-122 compromised microglial chemotaxis and led to reduced restriction of AD pathology
Source: J Neuroinflammation. 2024 Jul 2;21:167. doi: 10.1186/s12974-024-03162-z (PMC11218311; doi:10.1186/s12974-024-03162-z)
Supplement: Supplementary file 1 — Supplementary Material 1 [file 12974_2024_3162_MOESM1_ESM.docx]

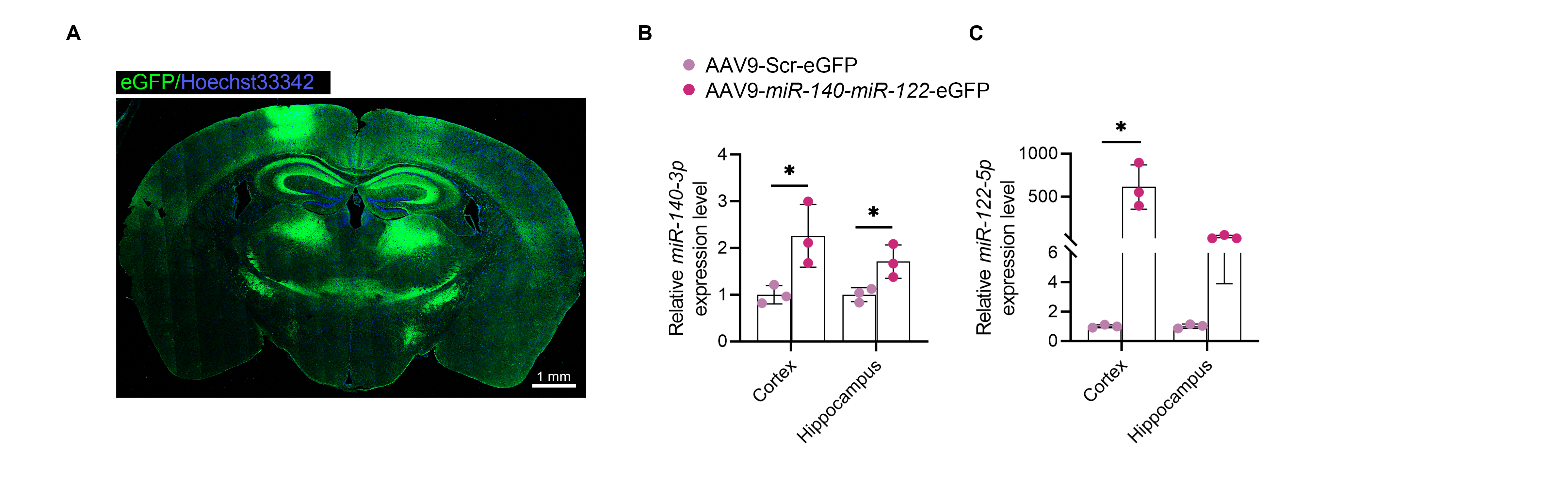


**Figure S1. Widespread neuronal cell infection after intracerebroventricular injection of recombinant AAV9.** (**A**). The representative confocal scanning images showing the widespread infection of AAV9-*miR-140*-*miR-122*-eGFP in the brain of an APP/PS1 mouse 2 month after a bilateral stereotactic injection (4×10^10^ genome copies in each side) into lateral ventricles (posterior: +0.3 mm; mediolateral: ± 1.0 mm, dorsal: -2.0 mm). The scale bar was 1 mm.

(**B** and **C**). Results of quantitative RT-PCR, showing the significantly increased expression of *miR-140* (**B**) and *miR-122* (**C**) in the cortex as well as in the hippocampi of APP/PS1 mice transfected with AAV9-*miR-140*-*miR-122*-eGFP, compared with that of the mice transfected with AAV9-Scr-eGFP. The results were presented as means ± SD (n = 3). **p* < 0.05. Statistical analyses were performed using two-tailed unpaired *Student’s t*-test.


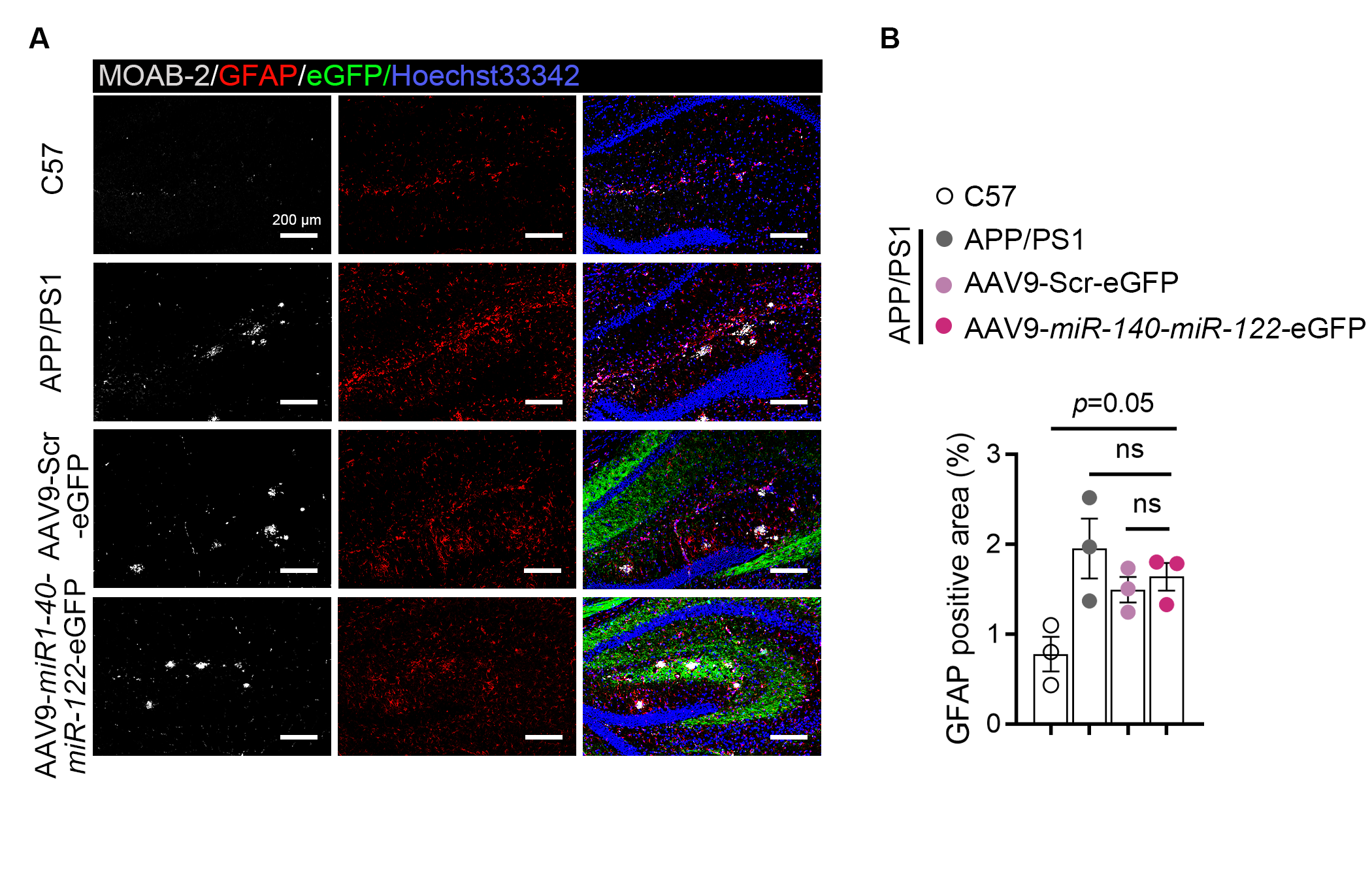


**Figure S2. Unaffected astrogliosis in the hippocampi of APP/PS1 mice overexpressing *miR-140* and *miR-122*.**  (**A**). Immunohistochemical analysis of Aβ deposition and astrogliosis using anti-Aβ antibody (MOAB-2) and anti-GFAP in the DG subregions on coronal sections of the hippocampus. Scale bar: 200 μm. (**B**). Quantification of the percentage of GFAP positive area showed increased astrogliosis in the hippocampi of APP/PS1 mice, compared with that of the control wide type C57 mice. Influences of astrogliosis was not observed in the hippocampi of APP/PS1 mice transfected with AAV9-*miR-140*-*miR-122*-eGFP, compared with that of the APP/PS1 mice transfected with AAV-Scr-eGFP. Results of three consecutive sections of three independent biological replicates were presented as means ± SEM. Statistical analyses were performed using One-way ANOVA.

**Table S4. Sequence of oligonucleotides for GV412 vector**

| **Sequence of oligonucleotides for GV412 vector** |
| --- |
| 5’-TGCTGCCCGACAACCACTACCTGAGCACCCAGTCCGCCCTGAGCAAAGACCCCAACGAGAAGCGCGATCACATGGTCCTGCTGGAGTTCGTGACCGCCGCCGGGATCACTCTCGGCATGGACGAGCTGTACAAGGCTAGCTAACTGGAGGCTTGCTGAAGGCTGTATGCTGTACCACAGGGTAGAACCACGGGTTTTGGCCACTGACTGACCCGTGGTTACCCTGTGGTACAGGACACAAGGCCTGTTACTAGCACTCACATGGAACAAATGGCCCGGGCCCTTTAAACTGGAGGCTTGCTGAAGGCTGTATGCTGTGGAGTGTGACAATGGTGTTTGGTTTTGGCCACTGACTGACCAAACACCTGTCACACTCCACAGGACACAAGGCCTGTTACTAGCACTCACATGGAACAAATGGCCCACCGGTTATCGATAATCAACCTCTGGATTACAAAATTTGTGAAAGATTGACTGGTATTCTTAACTATGTTGCTCCTTTTACGCTATGTGGATACGCTGCTTTAATGCCTTTGTATCATGCTATTGCTTCCCGTATGGCTTTCATTTTCTCCTCCTTGTATAAATCCTGGTTGCTGTC-3’ |

The sequences for cloning site were underlined. The sequences for mature *miR-140-3p* and *miR-122-5p* were red.
