# Supplementary figures and images for: Dysregulated expression of miR-140 and miR-122 compromised microglial chemotaxis and led to reduced restriction of AD pathology
Source: J Neuroinflammation. 2024 Jul 2;21:167. doi: 10.1186/s12974-024-03162-z (PMC11218311; doi:10.1186/s12974-024-03162-z)

Figure 2

2D

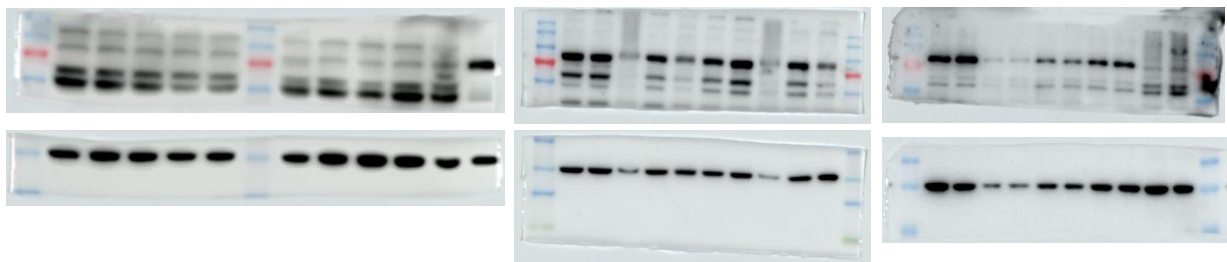

2E

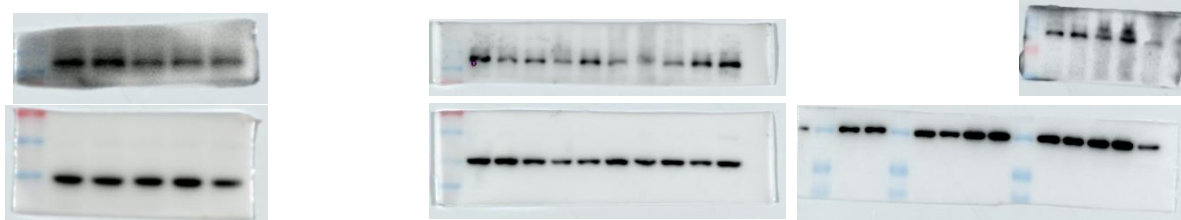

2F

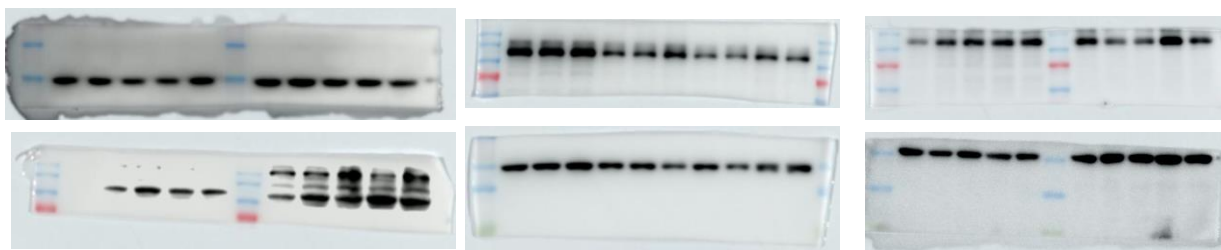

2G

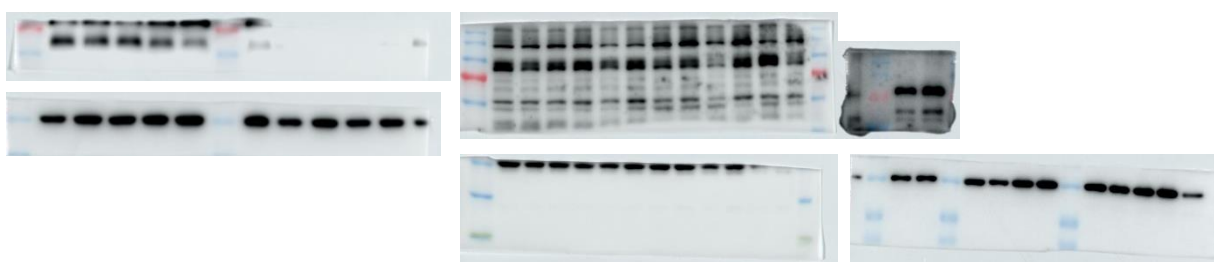

2H

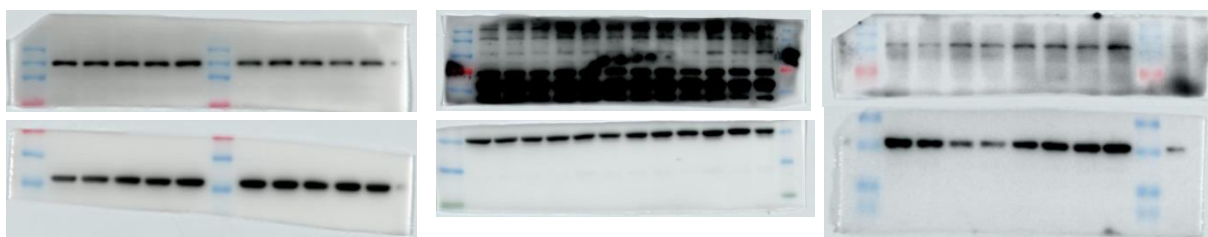

2I

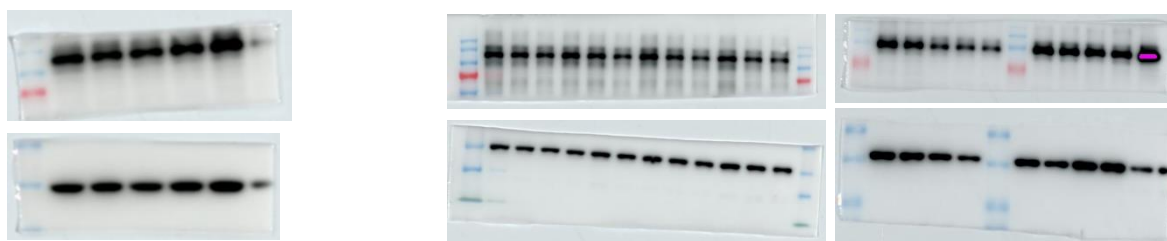

Figure 3

3G

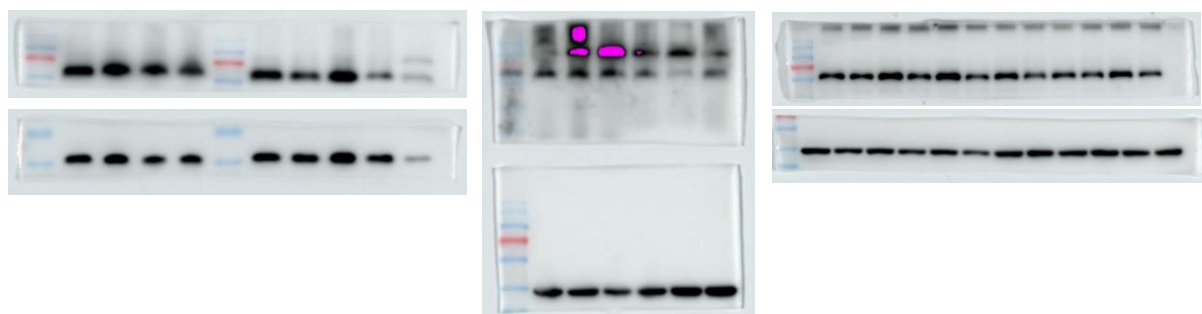

3H

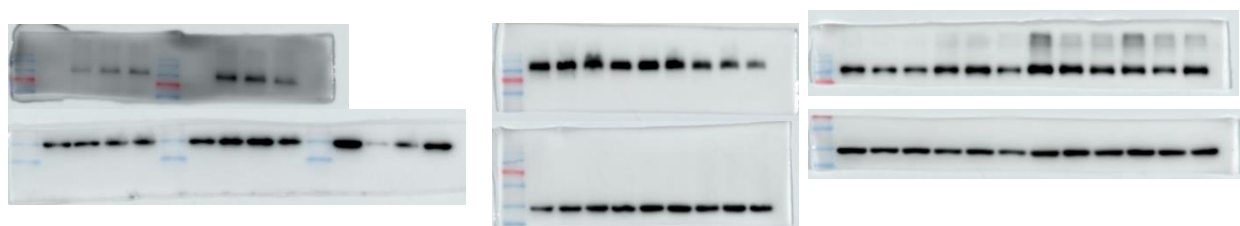

3I

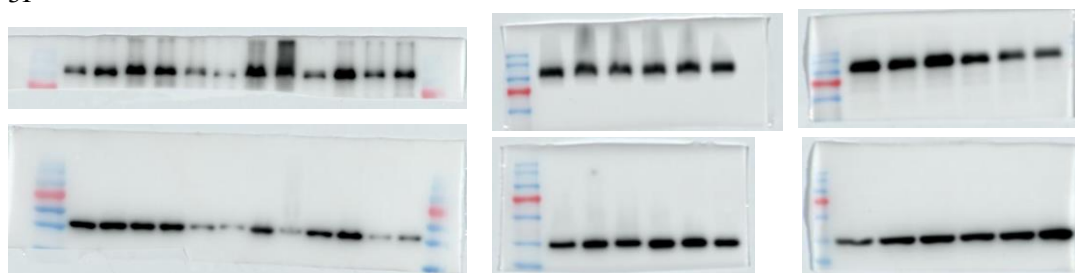

Figure 4

4F

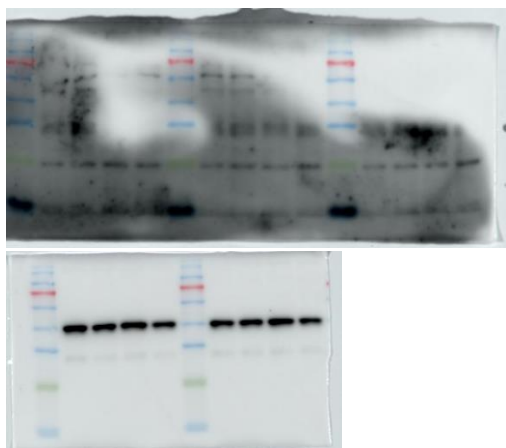

4G

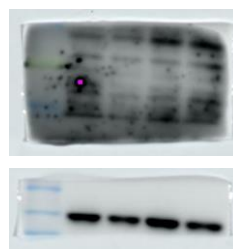

4k

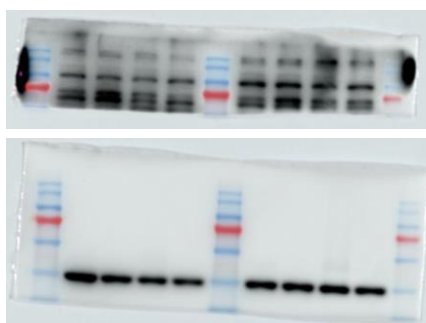

4L

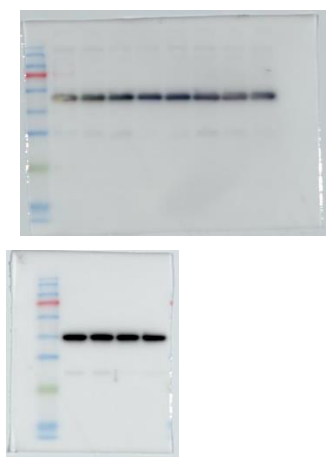

4M

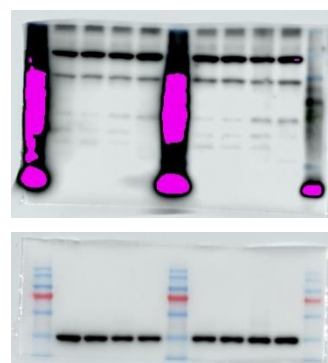

Supplement: Supplementary file 5 — Supplementary Material 5 [file 12974_2024_3162_MOESM5_ESM.pdf]
